# Distributed Temporal Coding of Visual Memory Categories in Human Hippocampal Neurons Revealed by an Interpretable Decoding Model

**DOI:** 10.1002/advs.202502047

**Published:** 2025-07-08

**Authors:** Xiwei She, Bryan J. Moore, Brent M. Roeder, George Nune, Brian S. Robinson, Brian Lee, Susan Shaw, Hui Gong, Christianne N. Heck, Gautam Popli, Daniel E. Couture, Adrian W. Laxton, Vasilis Z. Marmarelis, Samuel A. Deadwyler, Charles Liu, Theodore W. Berger, Robert E. Hampson, Dong Song

**Affiliations:** ^1^ Department of Biomedical Engineering Viterbi School of Engineering University of Southern California Los Angeles CA 90089 USA; ^2^ Wake Forest Institute for Regenerative Medicine Wake Forest University School of Medicine Winston‐Salem NC 27101 USA; ^3^ Department of Neurological Surgery Keck School of Medicine University of Southern California Los Angeles CA 90089 USA; ^4^ Neurorestoration Center University of Southern California Los Angeles CA 90033 USA; ^5^ Department of Neurology Rancho Los Amigos National Rehabilitation Hospital Downey CA 90242 USA; ^6^ Department of Neurology Wake Forest University School of Medicine Winston‐Salem NC 27101 USA; ^7^ Department of Neurosurgery Wake Forest University School of Medicine Winston‐Salem NC 27101 USA

**Keywords:** human hippocampus, memory category, memory decoding model, neuronal spike, spatio‐temporal code

## Abstract

The hippocampus is crucial for forming new episodic memories. While its role in encoding spatial and temporal information (where and when) is well understood, how it encodes objects (what) remains unclear due to the high dimensionality of object space. Rather than encoding each object separately, the hippocampus may encode object categories to reduce complexity. Here, an experimental‐modeling approach to investigate how the hippocampus encodes visual memory categories in humans is developed. Spikes are recorded from hippocampal CA3 and CA1 neurons in 24 epilepsy patients performing a delayed match‐to‐sample task involving five image categories. An interpretable memory decoding model is employed to decode memory categories from hippocampal spiking activity and identify the spatio‐temporal characteristics of hippocampal encoding. Using this model, the optimal temporal resolutions for decoding each visual memory category per neuron are estimated. Results indicate that visual memory categories can be decoded from hippocampal spike patterns, supporting the presence of category‐specific coding. Hippocampal neuron ensembles encode memory categories in a distributed manner, akin to a population code, while individual neurons use a temporal code. Additionally, CA3 and CA1 neurons exhibit similar and redundant memory category information, likely due to strong and diffuse feedforward synaptic connections from CA3 to CA1 regions.

## Graphical abstract

This study examines how the human hippocampus encodes visual memory categories. By decoding spiking activity from CA3 and CA1 neurons using an interpretable model, it reveals that category‐specific representations emerge from distributed temporal coding. These findings shed light on how complex object information is organized in memory through single‐neuron activity and ensemble dynamics.

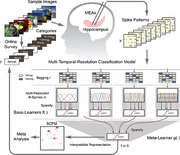

## 1 Introduction

The hippocampus is a brain region critical for the formation of new episodic memories.^[^ ^1^, ^2^, ^3^ ^]^ Impairment to the hippocampus due to diseases or injuries leads to profound memory deficit.^[^ ^4^, ^5^, ^6^, ^7^ ^]^ Therefore, hippocampal neurons are naturally positioned to encode episodic memory‐related information such as what (object), when (time), and where (space) of past events. While the encoding of spatial (“where”)^[^ ^8^, ^9^, ^10^, ^11^, ^12^ ^]^ and temporal (“when”)^[^ ^13^, ^14^ ^]^ information by hippocampal neurons is relatively well characterized‐largely due to the low dimensionality of these domains (e.g., 2D space, 1D time)–how high‐dimensional object information (“what”) is represented remains less understood.

Indeed, the space of objects has nearly infinite dimensions. It is infeasible for the hippocampus to encode every individual object separately. One possible strategy is to encode categories and/or features of objects to reduce the dimensionality of the object space. This is supported by rodent,^[^ ^15^, ^16^ ^]^ nonhuman primate (NHP),^[^ ^17^ ^]^ and human studies,^[^ ^18^, ^19^, ^20^, ^21^ ^]^ showing that hippocampal neurons increased their firing rates in response to visual stimuli (places and/or images) within the same categories during a memory task.

These previous studies have primarily investigated hippocampal encoding of objects at the single‐neuron level by analyzing averaged activity patterns.^[^ ^17^, ^18^, ^19^, ^20^, ^21^, ^22^ ^]^ Perievent histograms of neurons are calculated from spike patterns across many trials using a preselected bin size (temporal resolution of encoding). Category‐specific neurons were then identified based on the correlation between their perievent histograms and image categories. Although this approach has provided valuable insights into hippocampal neuronal encoding,^[^ ^19^, ^20^, ^21^, ^22^, ^23^, ^24^, ^25^ ^]^ the precise nature by which the hippocampus neuronal ensemble encodes category‐specific information through its spatio‐temporal patterns of spikes is not fully characterized. For example, it is unclear how the same hippocampal neuron encodes multiple categories or whether hippocampal neurons encode categories using the same or different temporal resolutions.

**Figure 1:**
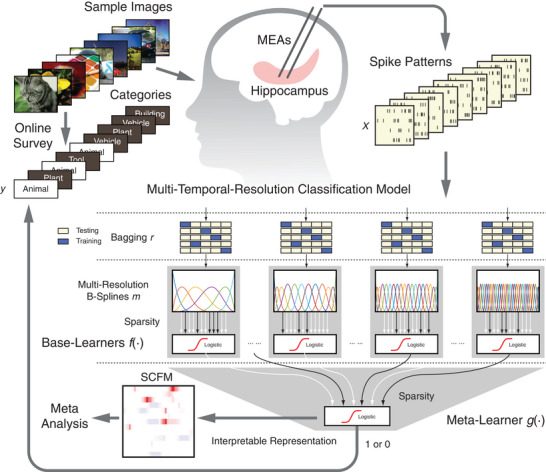
Decoding visual memory categories from spatio‐temporal patterns of spikes recorded in the human hippocampus using an ensemble multi‐temporal‐resolution classification model. This model provides interpretable model representations of spatio‐temporal characteristics of spike patterns for encoding specific memory categories.

To answer these questions, we developed and applied a combined experimental‐modeling approach to quantitatively investigate hippocampal encoding of visual memory categories in human subjects using an interpretable decoding model. Hippocampal CA3 and CA1 spikes were recorded from epilepsy patients performing a visual delayed match‐to‐sample (DMS) task involving multiple categories of images. To investigate how visual memory categories are encoded in hippocampal spike patterns, we applied an ensemble classification model operating across multiple temporal resolutions, which we previously developed and validated.^[^ ^35^ ^]^ This model enables the decoding of high‐dimensional spatio‐temporal spike patterns and yields interpretable representations of their spatio‐temporal structure (**Figure** **1**). Notably, this decoding task presents a statistically underdetermined problem, as the number of spatio‐temporal features extracted from neural recordings far exceeds the number of available trials per subject. To mitigate this, the model incorporates regularization and ensemble learning techniques such as bagging and stacking. Results show that visual memory categories can be decoded from hippocampal spike patterns with significantly above chance‐level accuracies, which strongly supports the existence of category‐specific coding in the human hippocampus. Hippocampal neuron ensembles encode visual memory categories in a distributed manner, similar to a population code, while each neuron encodes visual memory categories with a temporal code. In addition, hippocampal CA3 and CA1 neurons contain similar and redundant information about visual memory categories, possibly due to the strong feedforward synaptic pathway between the two regions.^[^ ^26^ ^]^ We believe this study advances the understanding of the neural code underlying memory category representation and provides a foundational basis for future development of stimulation applications in hippocampal neuroprosthetics.^[^ ^27^ ^]^

## 2 Experimental Section

### 2.1 Human Hippocampal Recording

**Figure 2:**
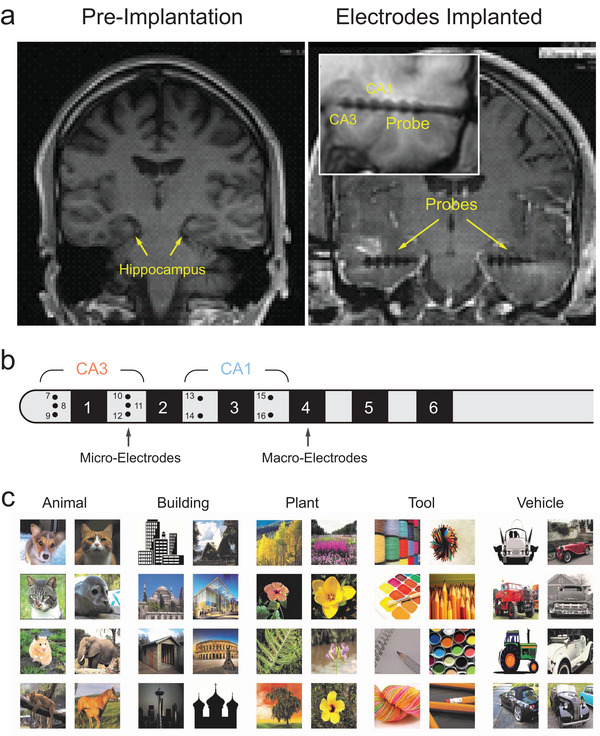
Human experimental paradigm. a) 3T MRI showing pre‐implantation hippocampal structures and post‐implantation electrode locations in one subject. The bulges visible in the probe are the location of the “macro” electrode sites. Inset: zoomed‐in view of the probe in the hippocampus. b) Layout of the micro‐macro probe containing 6 macro‐electrodes and 10 micro‐electrodes. Six and four micro‐electrodes were implanted in the CA3 and CA1 regions, respectively. c) Sample Images of the five memory categories used in the DMS task.

All participants were diagnosed with refractory focal epilepsy and underwent intracranial depth electrode implantation for seizure localization and monitoring (**Figure** **2**a). The number and placement of electrodes were determined entirely by the clinical team, based on clinical criteria. Typically, each subject received 1–4 FDA‐approved Ad‐Tech (Medical Instrumentation Corporation or PMT Corporation) “Macro‐Micro” depth electrodes. The “macro”‐electrodes recorded low‐frequency signals such as clinical electroencephalography (EEG), while the “micro”‐electrodes recorded higher‐frequency signals like single‐unit activity (spikes). Throughout the experiment, brain signals were recorded using the Blackrock Cervello system concurrently with a clinical EEG recording system. Electrode placement was performed intraoperatively using either a stereotactic headframe or a frameless stereotactic system to align the electrodes perpendicularly to the long axis of the hippocampus, targeting both CA3 and CA1 regions. Post‐operative MRI and electrophysiological recordings confirmed the accuracy of the electrode placements (Figure 2a) using the same techniques, which were previously demonstrated for validation of electrode placement.^[^ ^28^ ^]^ In this study, each probe contained 10 micro‐electrodes, with 6 in the CA3 region and 4 in the CA1 region (Figure 2b). Spikes were obtained by isolating single‐unit action potential waveforms from continuous recordings through online (Blackrock Cervello system) and offline (Plexon Offline Sorter) spike sorting procedures. A quality assessment of recorded neuronal signals can be found in Section S1 (Supporting Information).

All procedures were reviewed and approved by the Institutional Review Board of the University of Southern California and Wake Forest University in accordance with the National Institute of Health. All subjects provided voluntary written informed consent prior to participation in this study. Experiments were performed at Keck Hospital of the University of Southern California (Keck), Rancho Los Amigos Rehabilitation Center (Ranco), and Wake Forest Baptist Medical Center (Wake).

### 2.2 Behavioral Task

**Figure 3:**
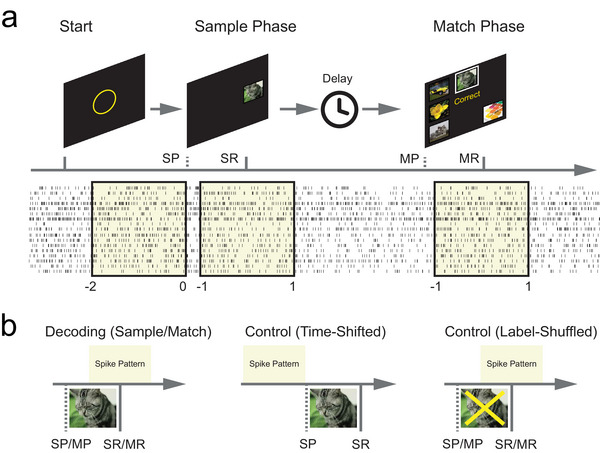
Behavioral tasks and decoding cases are designed for decoding memory category information. a) DMS task paradigm. SP: Sample Presentation; SR: Sample Response; MP: Match Presentation; MR: Match Response. b) decoding cases and control cases in the modeling.

Subjects were given a recovery period of 1–2 days from the anesthesia. Patients remained in the Intensive Care Unit (ICU) of Keck and Rancho and the Epilepsy Monitoring Unit (EMU) of Wake during the duration of their time in the hospital, where all behavioral tasks were performed. Subjects performed the memory‐dependent DMS task with a touch‐screen computer while sitting either in a bed or in a chair next to the bed in the ICU/EMU. Each DMS trial commenced with the display of a focus ring in the center of a touch screen (**Figure** **3**a). Subjects were instructed to click on the focus ring to initiate the Sample phase. In the Sample phase, a sample image was presented at a randomly selected location on the touch screen (Sample Presentation event). Subjects were instructed to remember this sample image and subsequently click on it to trigger a Sample Response event. Upon clicking, the sample image disappeared, and the screen remained blank for 3–5 s (Delay Phase). Following the Delay Phase, multiple images, including the sample image, were simultaneously presented on the touch screen at different locations. Subjects were instructed to select and click on the sample image based on their memories to generate a correct Match Response. One DMS task session consisted of 100–150 trials. Each subject completed 1–2 sessions of the DMS task.

Sample images (*n* = 500) were obtained from the internet. Five main categories (“Animal”, “Building”, “Plant”, “Tool”, and “Vehicle”) of images were included in the DMS task (Figure 1c). Note that “Building” stimuli depicted single, standalone structures without background context, and were treated as discrete objects rather than as full scenes or navigational environments. Additionally, due to the diversity within the “Tool” category, images in this group were limited to handicraft consumable items such as pencils, pens, markers, crayons, thread, yarn, and similar objects. Volunteers labeled the categories (1: in category, 0: not in category) of these images using an online survey. Each image was labeled 56 times on average. Only images with high scores (>0.9) in their respective categories were included. Binary labels of categories of images were used as the output signal of the memory decoding model.

### 2.3 Decoding Cases

Based on the behavioral task, two decoding cases and two negative control cases were designed (Figure 3b).

In the two decoding cases, spatio‐temporal patterns of spikes during “Sample Response” (memory encoding) events and “Match Response” (memory retrieval) events were used as model inputs, respectively. Binary (1 or 0) labels of the five memory categories of the sample images were used as model outputs. The objective of these two cases was to assess the model's ability to decode memory categories when memory was being encoded and retrieved, respectively.

In the first control case (Time‐Shifted), the time windows of spike patterns were shifted to be before the “Sample Presentation” events, so the input spike patterns contain no information about the output memory categories. In the second control case (Label‐Shuffled), model outputs were randomly shuffled across samples, thereby disrupting any potential correlation between them and the input spike patterns. These two negative control cases were included to verify that the model does not overfit the data, thereby ensuring the reliability of the decoding results.

### 2.4 Memory Decoding Model

The memory decoding model decodes spatio‐temporal patterns of spikes into binary visual memory category labels (Figure 1). It consists of two layers of learners (classifiers).

In its first layer, a bank of base learners (*L*1‐regularized logistic regression classifiers)^[^ ^29^ ^]^ extracts spatio‐temporal features from spike patterns with a wide range of temporal resolutions using B‐spline functions with different numbers of knots.^[^ ^30^, ^31^ ^]^ Each base‐learner, using a single temporal resolution, acts as a weak classifier on its own. *L*1‐regularization is used to reduce feature dimensionality and yield sparse estimation of model coefficients.^[^ ^29^ ^]^ The bagging method is adopted to reduce estimation variances by partitioning the data into multiple replicas and estimating multiple copies of the base learners with these replicas (ensemble classifier).^[^ ^32^ ^]^

In its second layer, a meta‐learner combines outputs from the base learners, each operating at a single temporal resolution, into an ensemble model using another *L*1‐regularized logistic regression classifier. It fuses multiple temporal resolutions into the model to classify spatio‐temporal patterns of spikes into memory category labels. It renders the model multi‐temporal resolution and a stronger classifier.

The model can be mathematically expressed as

(1) $$y=g\left(f_{r}^{\left(m\right)}\left(X\right)\right)$$

where *X* is the input spike pattern, *y* is the output memory label, *f*(·) is the base‐learner, *g*(·) is the meta‐learner, *m* is the number of B‐spline knots controlling the temporal resolutions, *r* is the index of the bagging replica. Given a set of B‐spline knots, the temporal resolution of each base‐learner is *M*/(*m* + 1), where *M* is the length of the decoding window (2 seconds in this study).

Nested cross‐validation is applied throughout the estimations to prevent overfitting.^[^ ^33^, ^34^ ^]^ Model coefficients are estimated using training data. Hyperparameters are optimized using validation data. Model performance is evaluated with test data that are held out from training and validation data.

This model was extensively evaluated using both synthetic and rodent data.^[^ ^35^ ^]^ Specifically, synthetic data results indicate that the model could identify optimal temporal resolutions by appropriately assigning weights to base learners with different resolutions. It can faithfully recover the ground truth temporal resolutions and firing probability intensity functions of the model neurons. When applied to the hippocampal spiking data recorded from rats performing a memory‐dependent delayed nonmatch‐to‐sample task, the model highly accurately decodes spatial memory information.

One important advantage of this model is its interpretability. It generates sparse classification functional matrices (SCFMs) representing the spatio‐temporal characteristics of hippocampal spike patterns most relevant to classification. SCFMs are calculated by nonlinearly integrating the predictions of the base‐learners using the meta‐learners as

(2) $$\begin{array}{c} F^{\prime }\left(n,\tau \right)={\left\{\;\;1+\exp\left(\;\;-{w^{\prime }}_{0}-\sum_{m=1}^{Q}\left[\sum_{j=1}^{J}\;b_{j}^{m}\left(\tau \right)w\left(n,j\right)\;\right]w^{\prime }\left(m\right)\;\right)\;\right\}}^{-1} \end{array}$$

where $b_{j}^{m}\left(\tau \right)$ are the B‐spline basis functions of each base‐learner; *w* are the model coefficients of base learners *f*(·); *J* is the total number of B‐spline knots used in a specific base‐learner. *w*′ are the model coefficients of the meta‐learner. *Q* is the total number of base learners. Note that the baseline probability is computed as $B_{j}^{m*}\left(\tau \right)={\left[1+exp\left(-w_{0}\right)\right]}^{-1}$ when $b_{j}^{m}\left(\tau \right)=0$. Regions where $b_{j}^{m}\left(\tau \right)$ exceeds $b_{j}^{m*}$ indicate areas where spikes increase the probability of belonging to a specific label, whereas regions with values below $b_{j}^{m*}$ indicate a decrease in this probability. More methodological details of the model can be found in Section S2 (Supporting Information) and a previous publication.^[^ ^35^ ^]^

When decoding a specific memory category, having spikes in SCFM regions with positive values (i.e., regions with SCFM value *exceed* the baseline $b_{j}^{m*}$) increases the probability of the pattern belonging to the decoded category. By contrast, having spikes in negative regions (i.e., regions with SCFM value *below* the baseline $b_{j}^{m*}$) of the SCFM decreases this probability. This allows for a direct quantification of specific spatio‐temporal regions that maximize the differences between patterns of the decoded category and non‐decoded patterns.

Model performance is assessed using the Matthews correlation coefficient (MCC), which effectively handles imbalanced data. The MCC is calculated from the confusion matrix components, such as true positives (TP), true negatives (TN), false positives (FP), and false negatives (FN), using below given formula

(3) $$\begin{array}{c} MCC=\frac{TP\times TN-FP\times FN}{\sqrt{\left(TP+FP\right)\times \left(TP+FN\right)\times \left(TN+FP\right)\times \left(TN+FN\right)}} \end{array}$$

This coefficient provides a value between −1 and 1, where −1, 0, and 1 indicate opposite, random, and perfect classification, respectively. Models that predict all outputs as a single class (i.e., all 1 or all 0), or randomly (chance level) will result in an MCC of 0. Additional metrics, such as Informedness and Markedness, were also calculated to better quantify the modeling performances.

Permutation feature importance (PFI) analysis quantifies the contribution of individual or groups of features to the predictive power of a model by evaluating the impact of their random alteration.^[^ ^34^, ^36^ ^]^ The process involves permuting each input feature or each group of input features independently and observing the resultant increase in model loss (e.g., cross‐entropy^[^ ^37^ ^]^ in this study). This increase is a direct indicator of the feature's importance; a significant rise in model loss suggests a high dependency of the model on that feature for accurate predictions. Thus, the importance of each feature is assessed based on the degree to which randomizing the feature degrades model performance.

The entire modeling procedure for each subject was achieved in tens of hours by using a parallel computing strategy.^[^ ^38^ ^]^

### 2.5 Supplementary Analyses

To evaluate whether decoding performance relied on meaningful spatio‐temporal patterns, two additional control tests based on surrogate spikes were implemented. In the first control, the spike train of each neuron was circularly shifted by a random offset (with wrapping), preserving firing rate and inter‐spike interval distribution while disrupting the temporal coordination across neurons. In the second control, each spike within a trial was independently jittered by a random offset sampled uniformly from −20 to +20 ms, maintaining overall firing rates but disrupting the fine temporal precision of spike timing. Detailed methodology can be found in Section S4 (Supporting Information).

In addition, to better evaluate the advantage of incorporating multi‐temporal resolution, four rate‐coding based models, each using a different fixed bin size: 20, 50, 100, and 2000 ms (the full decoding window) were implemented. The modeling performance of the multi‐temporal‐resolution model was compared with these rate‐coding‐based models in Section S5 (Supporting Information).

## 3 Results

### 3.1 Human Subjects

Twenty‐four patients with medically refractory focal epilepsy and mild‐to‐moderate memory abnormalities were enrolled in the study (**Table** **1**). The age of subjects ranged from 20 to 62 years, with an average age of 36.6 ± 12.2 years. The subjects comprised 13 males and 11 females, ensuring a balanced gender distribution. Among the 24 subjects, 11 had bilateral recordings on both the anterior and posterior regions of the hippocampus; 8 had bilateral recordings on the anterior region of the hippocampus; 5 had unilateral recordings on the anterior region of the hippocampus. The average number of recorded neurons was 30.0 ± 15.7 per subject. During the memory tasks, subjects completed an average of 149.5 ± 25.4 trials. Task performance was high (99.0% ± 2.2%), and our analyses focused on correct trials to ensure that the decoded neural signals reflected successfully encoded memory representations, while also maintaining a sufficient number of trials for reliable model training.

**Table 1:** Demographics and experimental characteristics for study participants.

Variable	Value
**Total number of subjects**	24
**Age [years]**	36.6 ± 12.2 (range: 20–62)
**Sex [M/F]**	13 / 11
**Recording Sites**	
– Bilateral anterior & posterior HC	11 subjects
– Bilateral anterior HC only	8 subjects
– Unilateral anterior HC only	5 subjects
**Number of neurons recorded per subject**	30.0 ± 15.7
**DMS Trials per subject**	149.5 ± 25.4
**Task performance [%]**	99.0 ± 2.2

^*^HC: hippocampus; DMS: delayed match‐to‐sample task.

### 3.2 Decoding Memory Categories Using a Multi‐Temporal‐Resolution Classification Model

We apply the memory decoding model to test whether hippocampal spiking activities contain visual memory category‐specific information. This model decodes memory categories (outputs) from hippocampal spatio‐temporal patterns of spikes (inputs) recorded at different phases of the memory‐dependent DMS task.

**Figure 4:**
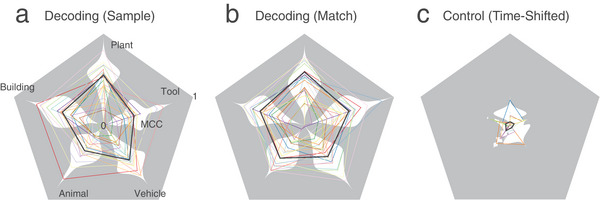
Categories of sample images can be decoded from spatio‐temporal patterns of spikes recorded during Sample Response and Match Response events of a delayed DMS task in human subjects (*n* = 24). Pentagon plots show the decoding performance of the five memory categories in a) Sample; b) Match; and c) Time‐Shifted decoding cases. Color lines: MCCs of individual subjects; Black thick lines: average MCCs across all subjects; White shades: distributions of MCCs within categories. Note that the Label‐Shuffle control case is omitted for clarity, as it yielded zero MCC values.

Results show that in both “Sample Response” and “Match Response” cases, the model yields significant classification accuracies, indicated by the Matthews correlation coefficients (MCCs) between true labels and predicted labels, in most categories and subjects (**Figure** **4**a,b). Average MCCs of the “Sample Response” case are 0.29 ± 0.16, 0.39 ± 0.23, 0.47 ± 0.19, 0.29 ± 0.16, 0.40 ± 0.19 for the five memory categories, respectively (Table S1, Supporting Information); note the chance‐level MCC = 0). In the “Match Response” case, MCCs of the five memory categories are 0.38 ± 0.17, 0.43 ± 0.18, 0.5 ± 0.15, 0.43 ± 0.18, and 0.36 ± 0.20, respectively (Table S2, Supporting Information). These results indicate that spatio‐temporal patterns of spikes during both memory encoding and memory retrieval periods contain image category information that can be decoded by the classification model.

In both control cases, the model yields near‐zero MCCs. The mean MCCs of the “Time‐Shifted” case (Figure 4c) for the five categories are 0.06 ± 0.11, 0.04 ± 0.09, 0.03 ± 0.10, 0.04 ± 0.11, and 0.03 ± 0.10, respectively (Table S7, Supporting Information). The mean MCCs of the “Label‐Shuffled” case are all 0. These results affirm that the classification model effectively avoids overfitting and decodes real memory‐related information from the hippocampal spiking activities.

Moreover, when tested on surrogate spike trains generated by either circular time shifting control or spike time jittering control, decoding performance dropped to near chance level (Tables S8–S11, Supporting Information). The original model significantly outperformed both control conditions across all subjects and categories (paired *t*‐test, *p* < 0.0001). Detailed results, including subject‐ and category‐level p values, percentile real‐data MCC values relative to surrogate distributions, are provided in Section S4 (Supporting Information).

### 3.3 Decoding Memory Categories Using Rate‐coding Based Classification Models

We implemented rate‐coding‐based models using several temporal bin sizes (20, 50, 100, and 2000 ms). These models are essentially “single‐resolution” models, as described in the manuscript and compared in our previous study.^[^ ^35^ ^]^ Each bin size corresponds to a specific temporal resolution, and the binning and averaging process effectively implements a zeroth‐order B‐spline. This differs slightly from the single‐resolution model described in our manuscript, which employs a third‐order B‐spline.

We evaluated these rate‐coding models on both Sample Response and Match Response decoding tasks and compared their performance to that of our multi‐temporal‐resolution model using statistical analyses. The results showed that the multi‐temporal‐resolution model significantly outperformed all rate‐coding models across all bin sizes (*p* < 0.001 for all paired comparisons). Among the rate‐coding models, those using 20, 50, and 100 ms bin sizes achieved significantly above‐chance decoding performance (MCC > 0). A general trend emerged whereby smaller bin sizes yielded better performance, suggesting that finer temporal granularity enhances decoding accuracy. In contrast, the model using a single 2000 ms bin (i.e., averaging firing rate across the entire decoding window) did not perform significantly better than chance (Figure S3 and Tables S12–S19, Supporting Information).

### 3.4 Spatio‐Temporal Distribution of Category Information in Hippocampal Spikes

The classification model used in this study is designed to be biologically interpretable.^[^ ^35^ ^]^ It characterizes the mapping between input spikes to output categories by explicitly representing it in the form of sparse classification functional matrices (SCFMs), which quantifies spatio‐temporal regions that maximize the differences between patterns of the decoded category and non‐decoded patterns (i.e., patterns of other categories). The SCFM has the same dimension as the spatio‐temporal pattern to be decoded. In an SCFM of a given category, zero‐valued (white‐baseline) areas do not contribute to the prediction of the memory category; positive (red‐*exceed* the baseline) areas represent spatio‐temporal regions where observing spikes increases the likelihood of the decoded category; negative (blue‐*below* the baseline) areas represent regions where spikes decrease the likelihood of the decoded category.

**Figure 5:**
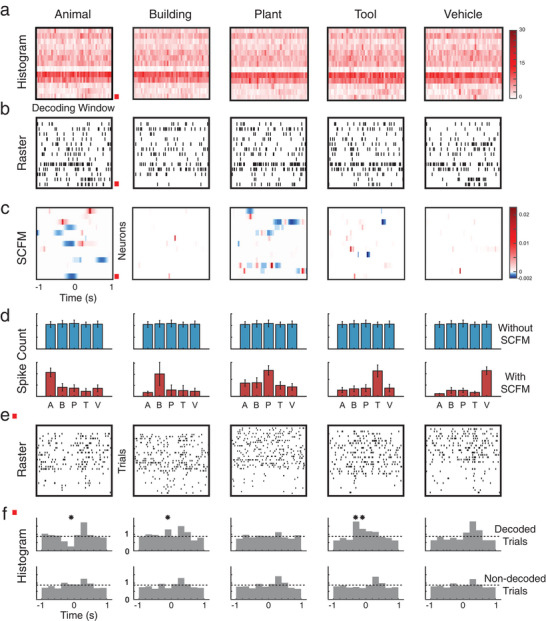
Spatio‐temporal distributions of category information in hippocampal spike patterns are revealed by the classification model. a) peri‐event histogram of spike patterns of the five categories during the Sample Response events. b) raster plots showing a single trial of the spatio‐temporal patterns of spikes during the Sample Response event. The five categories cannot be easily distinguished in either (a) or (b). c) SCFMs of the five categories in the classification model. The red box marks the neuron shown in (e) and (f). Based on the SCFMs, this neuron contributes to encoding the five categories. d) spike counts of each category with (top panel) and without (bottom panel) using SCFMs as masks. SCFMs reveal the spatio‐temporal regions of the spike patterns that encode the category information. e) spike raster plots of trials within each category of the neuron marked in (a), (b), and (c). f) peri‐event histograms of trials of the decoded category (top panel) and other (non‐decoded) categories (bottom panel) of this neuron. Dashed lines represent the baseline firing rates. Significant differences in firing rates between decoded categories and non‐decoded categories exist in time intervals consistent with the SCFMs (bins marked with asterisks, *p *< 0.05).

A representative model is presented to illustrate how the model decodes the spatio‐temporal patterns of spikes (**Figure** **5**). It is evident that the five categories cannot be easily distinguished from either the averaged spiking activities (Figure 5a) or the single‐trial spiking activities (Figure 5b). The SCFM highlights the spatio‐temporal regions most informative for category encoding (Figure 5c) and offers a direct means to examine the model's classification decisions. For example, there is no significant difference in the total spike counts within the patterns across the five categories (Figure 5d, top). However, after applying the SCFMs of the five categories identified by the model (Figure 5c) as masks to the spike patterns, i.e., calculating the inner product of the patterns and the SCFMs, these patterns show significant differences in spike counts within the SCFM regions across the five categories (Figure 5d, bottom). Note that this inner product operation projects the spike pattern onto the SCFM, producing a weighted summation across neurons and time points. This yields a scalar value that quantifies how well the current trial matches the learned spatio‐temporal pattern associated with a given category. Summing over this weighted matrix provides the model's predicted probability for the target memory label. As such, this result shows that the model identifies the spatio‐temporal regions critical for encoding the five categories of visual memories and further decodes these categories at the neuronal population level.

Based on the model, neurons with non‐zero values in their SCFM contribute to the encoding of categories. This can be verified at the single‐neuron level by comparing the firing patterns of the trials of the decoded category and non‐decoded categories of these neurons (Figure 5e). As shown in one example neuron, there are significant differences in firing rates between decoded categories and non‐decoded categories in time intervals consistent with the non‐zero regions of the SCFM (Figure 5f).

### 3.5 Sparseness of Spatio‐temporal Encoding of Categories

Encodings of the five categories are further characterized regarding spatial and temporal sparseness using classification models of all subjects (*n* = 24). Spatial sparseness refers to the proportion of neurons with only zero values in the SCFMs (i.e., no contribution to encoding) relative to the total number of neurons in each neuron ensemble. Temporal sparseness represents the proportion of zero‐valued time intervals of each neuron within its decoding window.

**Figure 6:**
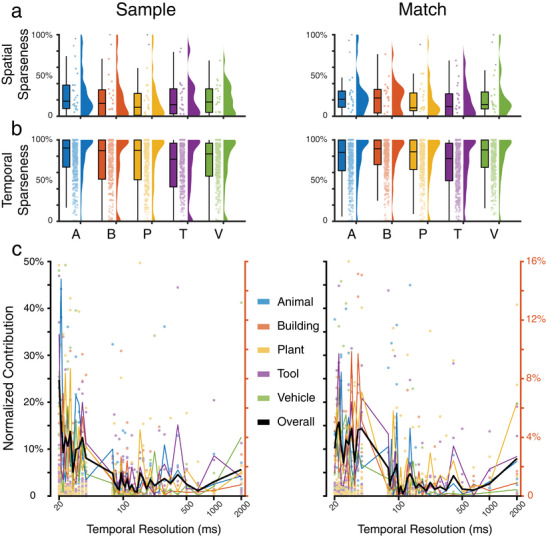
Sparseness of spatio‐temporal encoding of visual memory categories during Sample Response and Match Response events of the DMS task. a) spatial sparseness of all neuron ensembles (*n* = 24). b) temporal sparseness of all neurons (*n* = 721). Bars: mean sparseness; error bars: standard deviation (STD) of sparseness. c) Contribution of temporal resolutions to the encoding of visual memory categories. Colored dots (left *y*‐axis): contribution of temporal resolutions to the encoding of categories in each subject. Colored lines (right *y*‐axis): averaged contribution of temporal resolutions across all subjects (*n* = 24). Black line (right *y*‐axis): averaged contribution of temporal resolutions across all five categories. Left: Sample Response; Right: Match Response.

Results show that hippocampus neurons encode the five categories with similar levels of spatial and temporal sparseness (**Figure** **6**a,b, and **Table** **2**). Additionally, our results indicate that hippocampal neuron ensembles encode visual memory categories in a distributed manner (population coding), such as with lower spatial sparseness, while individual hippocampal neurons encode visual memory categories with temporal codes, such as with high temporal sparseness.

**Table 2:** Spatial and temporal sparseness of neural memory representation of five decoding memory categories across subjects (*n* = 24).

	Animal	Building	Plant	Tool	Vehicle
	Sample Response				
Spatial Sparseness (Mean ± STD)	28.2 ± 27.0%	24.1 ± 28.3%	21.1 ± 27.2%	21.5 ± 23.1%	20.1 ± 18.3%
Temporal Sparseness (Mean ± STD)	78.8 ± 26.1%	69.8 ± 35.3%	71.5 ± 33.4%	65.2 ± 34.9%	69.6 ± 33.5%
	Match Response				
Spatial Sparseness (Mean ± STD)	28.1 ± 27.3%	21.8 ± 18.7%	19.2 ± 21.5%	18.9 ± 20.2%	24.9 ± 27.6%
Temporal Sparseness (Mean ± STD)	75.8 ± 28.1%	77.9 ± 28.0%	74.4 ± 30.0%	69.8 ± 30.2%	76.9 ± 28.2%

### 3.6 Temporal Coding of Visual Memory Categories

The ensemble multi‐temporal‐resolution model incorporates a large range of temporal resolutions in its base learners, and the meta‐learner further chooses the optimal subset of base learners to decode the memory categories. Using the PFI analysis on the base learner of the model, we directly quantify the contribution of each temporal resolution to the encoding of visual memory categories by calculating the reduction of loss caused by permuting the features associated with each temporal resolution.

Results show that a wide range of temporal resolutions is utilized in encoding the five categories, with high temporal resolutions playing a more important role than low temporal resolutions (Figure 6c). Similar distributions of different temporal resolutions’ contributions are observed in all five categories during both the Sample Response and Match Response events of the DMS task. These results are consistent with the temporal sparseness results above and strongly suggest a temporal code, as opposed to a rate code, of hippocampal neurons in encoding visual memory categories.

### 3.7 Contribution of Hippocampal CA3 and CA1 Neurons to Encoding of Categories

**Figure 7:**
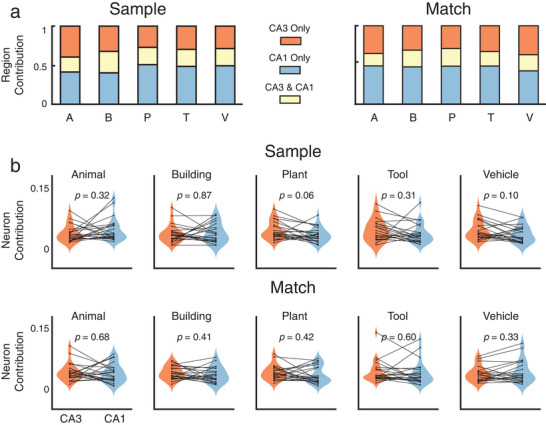
Contribution of hippocampal CA3 and CA1 to the encoding of visual memory categories during Sample Response and Match Response events of the DMS task. a) Contributions of the CA3 and CA1 regions. Red: unique contribution of the CA3 region; Yellow: redundant contribution shared by CA3 and CA1 regions; Blue: unique contribution of the CA1 region. b) averaged contribution of hippocampal CA3 and CA1 neurons to the encoding. Each dot represents one subject (*n* = 24). There is no significant difference between CA3 and CA1 neurons during both Sample Response and Match Response (paired *t*‐test).

To investigate how CA3 and CA1 neurons contribute to the encoding of memory categories, we further apply the PFI analysis to calculate the contribution of these neurons using their corresponding model features. First, the total contribution of each region, such as CA3 and CA1, is calculated as the reduction of the normalized cross‐entropy loss by permuting all features associated with all neurons in that region. Redundancy is calculated as the contribution shared by the two regions, such as the summation of the individual contributions of the two regions subtracts the total contribution of the two regions combined. The unique contribution of each region is then calculated as the individual contribution of each region, subtracting the redundancy. Results show that CA3 and CA1 neurons contribute similarly to encoding the five visual memory categories with a significant amount of redundancy (**Figure** **7**a). The unique contribution of CA3, the redundancy, and the unique contribution of CA1 averaged across all five categories are 31.5 ± 4.9%, 22.5 ± 3.0%, and 46.0 ± 4.9% in the Sample phase, and 33.0 ± 3.2%, 19.8 ± 2.7%, and 47.2 ± 2.7% in the Match phase.

To balance the unequal numbers of CA3 and CA1 neurons in each subject, the averaged contributions of individual CA3 and CA1 neurons are calculated by normalizing the overall contributions of CA3 and CA1 ensembles to encoding with the number of CA3 and CA1 neurons in each region. Results show no significant difference between CA3 and CA1 neurons in all five categories during both the Sample Response and Match Response events of the task (Figure 7b). These results indicate that CA3 and CA1 neurons contain similar information about visual memory categories, possibly due to the strong and divergent synaptic connections from CA3 to CA1 regions.^[^ ^26^ ^]^

## 4 Discussion

This study combines human electrophysiology and computational modeling to investigate how the hippocampus encodes visual memory categories with its spiking activities.

The first main finding of this study is that visual memory categories can be successfully decoded from hippocampal CA3 and CA1 spikes during both the Sample (encoding) and Match (retrieval) phases of the DMS task. This result confirms the pivotal role of the hippocampus in integrating sensory information, such as the “what” information of objects, for the formation of episodic memories. It strongly suggests that, due to the very high dimensionality of the object space, the hippocampus uses categories to reduce the dimensionality and parsimoniously encode object information using its neuronal ensembles.

In addition, we demonstrate that memory categories can be decoded from single trials of hippocampal ensemble spike patterns using a classification model, while the traditional decoding methods often rely on averaged neuronal firing patterns across many trials.^[^ ^17^, ^18^, ^19^, ^20^, ^21^, ^22^ ^]^ In this study, spatio‐temporal patterns of the neuronal ensemble are used as the input signals, allowing all neurons to contribute collectively to decoding output signals (i.e., memory categories). While each session included 20‐30 trials per category, our approach operates on high‐dimensional spatio‐temporal features, often numbering in the thousands, to fully capture the temporal dynamics of neuronal spiking activities. Sparse classifiers and ensemble learning techniques, such as bagging and stacking^[^ ^32^, ^39^ ^]^ are used to effectively avoid overfitting and enable decoding memory categories from very high‐dimensional input signals using relatively short data lengths. Importantly, this classification does not rely on a predefined temporal resolution in decoding. Instead, it incorporates a broad range of temporal resolutions in its base learners to extract multi‐scale temporal features from spike patterns. The optimal temporal resolutions are then determined using a data‐driven stacking method by the meta‐learner. This approach enables us to quantify the temporal resolution of decoding and address the key questions of which coding strategy, temporal or rate coding, is employed in the hippocampus. A key distinction between our approach and previous approaches is that we do not preselect neurons based on their firing rate or trial‐averaged activity patterns. Instead, our model analyzes the ensemble neuronal activity to identify important spatio‐temporal patterns contributing to memory categories. In addition, while some previous studies have also used modeling approaches to decode memory from neural signals,^[^ ^19^, ^20^, ^40^, ^41^ ^]^ these methods often use simple linear models, including a single, fixed temporal resolution.

Moreover, in contrast to many machine learning models that operate as “black boxes,” the classification model used in this study is highly interpretable. It offers intuitive representations of the spatio‐temporal characteristics of spike patterns, in the form of SCFMs, that are most relevant for decoding. This interpretability has been rigorously validated through both simulated data and experimental results from rodent studies,^[^ ^35^ ^]^ enabling further meta‐analysis of the SCFMs to gain additional insights into hippocampal memory encoding.

Through meta‐analyses of the SCFMs, we found that the hippocampus encodes memory categories in a spatially distributed yet temporally sparse manner. Across all five categories, ≈70%–80% of neurons were involved in encoding. However, within each neuron, only 20%–30% of the temporal window contributed to encoding these categories. This pattern is consistent with previous human studies on episodic memory using non‐visual stimuli such as words^[^ ^23^ ^]^ and aligns with recent human single‐neuron studies using visual stimuli,^[^ ^14^ ^]^ which similarly reported that a large proportion of hippocampal neurons respond to stimuli via brief, sharply timed bursts of activity during memory encoding. From a computational modeling perspective, such an encoding strategy may be optimal, as it balances two competing demands: maximizing the capacity to store diverse memories while minimizing energy consumption, which is positively correlated with the number of spikes.^[^ ^42^ ^]^ Furthermore, this temporally sparse activation supports a temporal coding strategy that has long been associated with hippocampal function.

The hippocampus is well established as a key structure for encoding episodic memory,^[^ ^1^, ^2^, ^3^ ^]^ which involves representing sequences of events and binding information across time. Two primary neural coding strategies have been proposed to explain how such information is encoded in spike trains: rate coding and temporal coding. Rate coding suggests that information is conveyed by the average firing rate of a neuron over a defined time window, typically on the order of hundreds of milliseconds. In this framework, stronger or more salient stimuli elicits higher firing rates. In contrast, temporal coding posits that the precise timing of individual spikes, often at the millisecond scale, carries meaningful information, enabling more efficient and higher‐capacity neural representations. Building on this framework, our study contributes new insights to the long‐standing debate over the relative importance of rate versus temporal coding strategies in neural information representation, particularly in the context of memory.^[^ ^2^, ^14^, ^24^, ^43^, ^44^, ^45^, ^46^ ^]^ While hippocampal neurons may employ both strategies,^[^ ^24^, ^45^ ^]^ our findings suggest that fine‐grained temporal resolution plays a more prominent role in encoding visual memory categories. This is also consistent with the temporal sparseness findings and suggests a temporal code, as opposed to a rate code, of hippocampal neurons in encoding visual memory categories.

This study also investigated the role of the hippocampal CA3 and CA1 regions in encoding memory categories. We found that neurons in both the hippocampal CA3 and CA1 regions contribute similarly and contain redundant information about memory categories. This is expected given the strong and diffuse synaptic projections from CA3 pyramidal neurons to CA1 pyramidal neurons.^[^ ^26^ ^]^ As a result, information in the CA3 region likely mirrors that in the CA1 region. In our previous studies on developing hippocampal memory prostheses, we demonstrated that the spiking activity of CA1 neurons can be accurately predicted from CA3 neuron activity using a multi‐input, multi‐output machine learning model.^[^ ^47^, ^48^, ^49^ ^]^ The presence of similar information in both regions enables the success of such a model, even when utilizing nonlinear dynamical models capable of capturing complex input‐output transformations.

In this study, the decoding model was primarily used as a tool to investigate how memory categories are encoded in spatio‐temporal spike patterns. While the model maps memory content to neural activity, it also holds potential for future applications in facilitating memory encoding through model‐based stimulation strategies. However, it is important to recognize that decoding and stimulation are not inherently bidirectional processes. Ensemble‐level decoding can tolerate some loss of single‐neuron specificity (e.g., using multi‐unit or unsorted spikes), whereas effective stimulation typically requires highly targeted interventions within neuronal circuits. Therefore, the development of future models capable of extracting spatio‐temporal structure from unsorted spikes would be highly valuable. Nonetheless, the current findings offer important insights into the spatio‐temporal nature of memory coding in the hippocampus and provide a foundational step toward the long‐term goal of developing hippocampal memory prostheses aimed at restoring or enhancing memory function.^[^ ^27^, ^31^, ^50^ ^]^

Several limitations of this study should be acknowledged, along with opportunities for future research. First, our experimental paradigm, the DMS task, was specifically designed to model memory encoding over short timescales. Accordingly, the form of memory examined in this study is best characterized as working memory, given the minimal delay between stimulus presentation and retrieval. As a result, our computational framework primarily addresses the mechanisms underlying short‐term memory encoding and decoding, without explicitly modeling processes related to episodic memory retrieval or memory forgetting. Given the brief retention interval (on the order of seconds), we assumed that memory traces remained stable with negligible decay. However, other processes such as episodic retrieval and long‐term forgetting, potentially involving distinct neural mechanisms such as pattern replay or memory degradation,^[^ ^51^, ^52^, ^53^, ^54^ ^]^ were not captured by the current framework. Future extensions could incorporate these processes by examining how memory representations evolve over longer timescales, ultimately providing a more comprehensive understanding of memory‐related neural dynamics in the human hippocampus.

Additionally, due to practical constraints inherent in clinical studies, we used a relatively small set of images spanning five largely independent categories. In everyday life, however, humans encounter a vast array of diverse objects belonging to numerous, often interrelated, categories. While our decoding model focused on distinguishing memory content at the categorical level, it remains possible that hippocampal neurons encode rich, high‐dimensional visual features, with category information emerging as a latent property. One consideration is that due to the brain's hierarchical coding architecture,^[^ ^55^, ^56^, ^57^ ^]^ objects with similar visual features may naturally cluster together, giving rise to category‐like representations. Our current study does not allow us to fully disentangle this possibility. Future investigations could address this by using stimuli that orthogonalize low‐level visual features and categorical labels. To fully explore memory encoding in naturalistic settings, future studies will also benefit from advanced neural recording techniques and modeling approaches capable of capturing the complexity of real‐world stimuli and neural responses.

## Conflict of Interest

The authors declare no conflict of interest.

## Author Contributions

X.S. performed methodology, formal analysis, data curation, investigate and visualized the project, worked with software, wrote the original draft, reviewed & edited the final manuscript. B.J.M. performed data curation, and investigate the project and wrote, reviewed and edited the final manuscript. B.M.R. performed data curation, and validation and investigate the project. G.N. investigates the project. B.S.R. performed data curation and investigate the project. B.L., S.S., and H.G. investigate the project. C.N.H. investigate and supervised the project. G.P., D.E.C and A.W.L investigate the project. V.Z.M. performed methodology. C.L. performed validation, and investigate the project, did project administration, and supervised the project. S.A.D. and T.W.B. did project administration and acquired funding. R.E.H. performed methodology and data curation, investigate and supervised the project, did project administration, and acquired funding. D.S. performed methodology, formal analysis, investigation, data curation, conceptualized the project, worked with software, visualized the project, and supervised the project, did project administration, acquired funding, and wrote the original draft, reviewed & edited the final manuscript.

## Supporting information



Supporting Information

## Data Availability

The complete set of raw and processed data utilized in this study is available upon request. The code, along with example data for demonstrating the modeling methodologies and reproducing figures in this manuscript, is publicly available at: https://github.com/neural‐modeling‐and‐interface‐lab/Human_Memory_Decoding.
