# Supplementary material for: Distributed Temporal Coding of Visual Memory Categories in Human Hippocampal Neurons Revealed by an Interpretable Decoding Model
Source: Adv Sci (Weinh). 2025 Jul 8;12(38):e02047. doi: 10.1002/advs.202502047 (PMC12520509; doi:10.1002/advs.202502047)
Supplement: Supplementary file 1 — Supporting Information [file ADVS-12-e02047-s001.docx]

**Supplementary Materials**

**Supplementary Material 1: Quality Assessment of Recorded Neural Signals**

To assess the quality and validity of the recorded neural signals, we present visualizations of neuronal activity using auto-correlograms from representative neurons (Supplementary Figure 1).


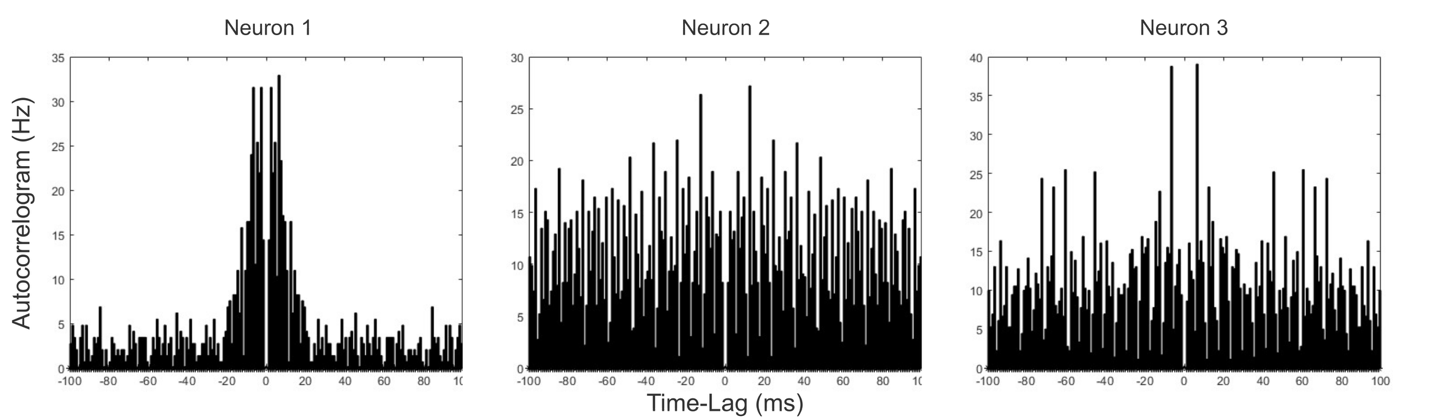


Supplementary Figure 1: Auto-correlograms (Hz) of representative neurons recorded from a single subject. The examples illustrate hallmark features of neuronal activity, including a refractory period, oscillatory behavior, and decaying correlations.

**Supplementary Material 2: Memory Decoding Model**

Model Configuration and Estimation

The memory decoding model has a two-layer structure.

The first layer contains a bank of base-learners $f\left( \cdot\right)$. Each base-learner utilizes a B-spline basis function $b_{j}$ with $m$ interior knots. B-splines, known for their piecewise polynomial structure with smooth transitions at specified interior knot points, allow for effective feature extraction. Spatio-temporal features of the spike patterns are generated as

$$z^{(n)}(j)=\sum_{\tau=0}^{M} b_{j}(\tau)x_{n}(\tau)$$

where $M$ represents the decoding window length (2 seconds in this study); $x_{n}$ represents the spike patterns for the $n^{th}$ neuron. The resulting spatio-temporal feature vector $z$ has a dimensionality of $1\times JN$, where $J$ is the total number of B-spline interior knots, and $N$ is the total number of input neurons. With a fixed degree $d$ (3 in this study) and a sequence of $m$ interior knots, $J$ is equal to $m+d+1$. In this study, the $m$ interior knots are evenly distributed in the decoding window [$-\frac{M}{2}$, $\frac{M}{2}$]. Thus, for a given set of interior knots, the temporal resolution of the B-spline basis is $M/(m+1)$.

The feature dimension is controlled by the B-spline resolution $m$. A larger $m$ leads to a higher feature dimension and a finer temporal resolution. By adjusting $m$, B-spline features can range from representing rate code to temporal code with varying temporal resolutions. In the first layer, a broad range of $m$ values are used to examine the temporal characteristics of neural activity, with each value corresponding to a distinct feature vector $z$ used to train a separate base-learner.

Each base-learner takes the form of a logistic regression classifier as

$$P(y=1|X)=[{1+exp(-w_{0}-\sum_{n=1}^{N} \sum_{j=1}^{J} w^{(n)}(j)z^{(n)}(j))]}^{-1}$$

$$P(y=0|X)=1-P(y=1|X)$$

Since the number of features (i.e., $JN$) is large (can exceed the number of samples), *L*1-regularized estimation is used to obtain sparse base-learner coefficients $w$ to avoid overfitting. The minimizer $S$ is defined as

$$S(w)= -l(w)+\lambda(\sum_{n=1}^{N} |w^{(n)}|)$$

where -$l$ represents the negative log-likelihood

$$-l(w)=\sum_{i=1}^{I} [y(i)*logP(y(i)=1|X)+(1-y(i)*log(1-P(y(i)=1|X)))]$$

$\lambda$ is the regularization hyper-parameter, with larger values leads sparser model estimations.

The second layer of the model is a meta-learner $g(\cdot)$ combining outputs of all base-learners with another *L*1-regularized logistic regression classifier as

$$P'(y=1|X)=[{1+exp(-w_{0}'-\sum_{q=1}^{Q} w'(q)P(q,y=1|X))]}^{-1}$$

where $q$ represents each of the $Q$ base-learner with a specific temporal resolution, and $w'$ denotes the meta-learner’s coefficients.

A bagging-based strategy is used to reduce the large variances in base-learner estimation due to the small sample size [1, 2]. Within the inner loop of the nested cross-validation, data are partitioned $R$ times to obtain $R$ replicas of training and validation datasets. A separate base-learner is trained on each replica. Hyper-parameters are optimized using all replicas, resulting in an ensemble estimate with reduced variance of the model coefficients. Due to the imbalanced data (much more 0s than 1s in labels), stratified *k*-fold nested cross-validation is used to ensure that each partition contains both 1 and 0 labels in both inner and outer loops [3, 4].

Model Interpretation

For each base-learner, sparse classification functional matrix (SCFM) is calculated as

$$F^{m}(n,\tau)=\sum_{j=1}^{J} b_{j}^{m}(\tau)w(n,j)$$

The SCFM has the same dimensions as the input spatio-temporal spike patterns $X$. It provides a functional map that relates spike patterns to the target classification label, and can be directly used to calculate the conditional probability of a label given $X$ as

$$\theta(\hat{y}(i)=1|X(t_{i}-\tau))=[{1+exp(-w_{0}-\sum_{n=1}^{N} \sum_{-\frac{M}{2}\leq\tau\leq\frac{M}{2}} F^{m}(n,\tau)x^{(n)}(t_{i}-\tau))]}^{-1}$$

For each neuron $n$, the $j^{th}$ B-spline basis function, modeled at a single resolution $m$, can be converted into probabilities using the associated model coefficients as

$$B_{j}^{m}(\tau)=[{1+exp(-w_{0}-b_{j}^{m}(\tau)w(n,j))]}^{-1}$$

It demonstrates how each B-spline basis function, in combination with the model coefficients, contributes to the conditional probability $\theta$. The baseline probability is computed as $B_{j}^{m}(\tau)=[{1+exp(-w_{0})]}^{-1}$ when $b_{j}^{m}(\tau)=0$. Regions where $b_{j}^{m}(\tau)$ exceeds $b_{j}^{m*}$ indicate areas where spikes increase the probability of belonging to a specific label (i.e., $\theta(\hat{y}(i)=1|X(t_{i}-\tau))$), whereas regions with values below $b_{j}^{m*}$ indicate a decrease in this probability.

With the meta-learner, an ensemble SCFM is generated by combining SCFMs from all base-learners, weighted by the second-layer coefficients as

$$F'(n,\tau)=[{1+exp(-w_{0}'-\sum_{q=1}^{Q} F^{q}(n,\tau)w'(q))]}^{-1}$$

Similarly, B-spline basis functions, represented as probabilities, can be calculated using the second-layer coefficients as

$$B_{j}'=[{1+exp(-w_{0}'-\sum_{k\neq q}^{Q} w'(k)B_{j}^{k*}-B_{j}^{q}(\tau)w'(q))]}^{-1}$$

Model Evaluation

The memory decoding model has been extensively tested with synthetic data and rodent data [5]. In the synthetic data test, spatio-temporal spike patterns are simulated with 30 neurons across 5 categories, chosen to reflect typical values encountered in human experiments. Each neuron had a 0.5 probability of having no Gaussian peaks (i.e., spatial sparseness is 0.5) and a 0.25 probability of exhibiting either 1 or 2 peaks in its probability intensity function (PIF). The Gaussian peaks had randomly assigned locations, widths, and intensities. To better illustrate the model’s ability to capture varying temporal resolutions, neurons were simulated with both low and high temporal resolutions, represented by the wide and narrow patterns (Supplementary Fig. 1A, B). For each category, 200 instances were generated.

Five memory decoding models are constructed for the five categories. Results show that these models can perfectly decode the population patterns into correct categories (MCC = 1 in all categories). SCFMs accurately recover PIFs with correct spatial sparseness and temporal resolutions (Supplementary Fig. 3A, C). SCFMs identify the spatio-temporal regions that are critical for predicting the categories (Supplementary Fig. 3D).


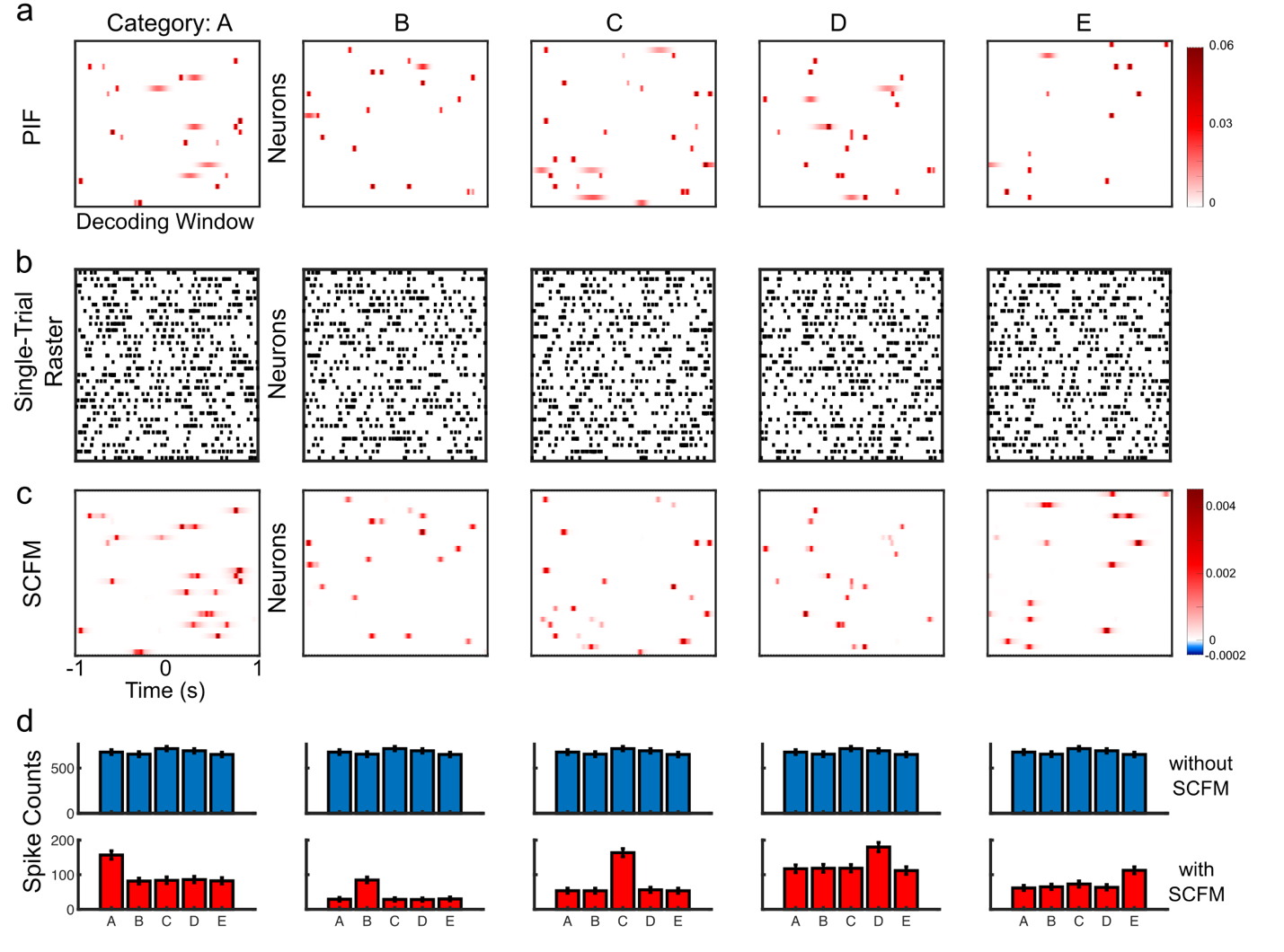


Supplementary Figure 2: Decoding population patterns. **a**: probability intensity functions (PIFs) of the five categories; **b**: raster plots of one example of spatio-temporal patterns of spikes simulated based on PIFs; **c**: SCFMs of double-layer decoding models. In a, b, and c, x-axis is the decoding time window (-2 sec to 2 sec); y-axis is indices of neurons. PIF, raster and SCFM all have the same dimensionality. **d**: spike counts of each category with (top panel) and without (bottom panel) using SCFMs as masks. SCFMs reveal the spatio-temporal regions of the spike patterns that encode the category information.

**Reference**:

[1] Breiman, L. (1996). Bagging predictors. Machine Learning. https://doi.org/10.1007/BF00058655

[2] Rindskopf, D. (1997). An introduction to the bootstrap - Efron,B, Tibshirani,RJ. Journal of Educational and Behavioral Statistics.

[3] Abma, B. (2009). Evaluation of requirements management tools with support for traceability-based change impact analysis. Master’s Thesis, University of Twente, Enschede.

[4] Diamantidis, N. A., Karlis, D., & Giakoumakis, E. A. (2000). Unsupervised stratification of cross-validation for accuracy estimation. Artificial Intelligence. https://doi.org/10.1016/S0004-3702(99)00094-6

[5] She, X., Berger, T. W., & Song, D. (2022). A double-layer multi-resolution classification model for decoding spatiotemporal patterns of spikes with small sample size. *Neural computation*, *34*(1), 219-254

**Supplementary Material 3: Performance of the Memory Decoding Models**

**Rationale**: This analysis presents detailed decoding performance results across all subjects and memory categories under the different modeling conditions.

**Data**: Twenty-four human subjects participated in the study. The decoding task involved five memory categories (Animal, Building, Plant, Tool, Vehicle).

**Methods**:

Modeling performance yield by the multi-temporal-resolution model was quantified by the Matthews Correlation Coefficients (MCC). Moreover, to provide a more comprehensive understanding of our model’s decoding capabilities, we also estimated our model performances using Informedness and Markedness, each providing a different perspective on classification performance.

*Statistical Comparison*: One-sample t-test was used to determine whether MCCs, Informedness, and markedness across subjects (n = 24) for each category (c = 5) at each control case was significantly greater than zero (chance level).

**Results**:

The model achieved significantly above chance-level performance largely across subjects and categories under both Sample Repones and Match Response condition (Supplementary Table 1-6), according to all three-evaluation metrics. Additionally, model performance under the Time-shifted Control case fell to near the chance level (Supplementary Table 7), indicating that the model did not overfit the data and instead captured meaningful, memory related information.

Supplementary Table 1: Modeling performances (MCC values) of the double-layer multi-temporal resolution model across all subjects (n=24) and categories in the **Sample Response** decoding case.

| **Subject ID** | **Animal** | **Building** | **Plant** | **Tool** | **Vehicle** |
| --- | --- | --- | --- | --- | --- |
| **S1** | 0.168 | 0.051 | 0.411 | 0.271 | 0.378 |
| **S2** | 0.300 | 0.345 | 0.559 | 0.231 | 0.452 |
| **S3** | 0.360 | 0.633 | 0.651 | 0.303 | 0.498 |
| **S4** | 0.144 | 0.368 | 0.490 | 0.101 | 0.135 |
| **S5** | 0.225 | 0.233 | 0.438 | 0.663 | 0.662 |
| **S6** | 0.721 | 0.747 | 0.526 | 0.330 | 0.607 |
| **S7** | 0.381 | 0.468 | 0.421 | 0.275 | 0.680 |
| **S8** | 0.141 | 0.367 | 0.569 | 0.362 | 0.222 |
| **S9** | 0.422 | 0.518 | 0.293 | 0.343 | 0.069 |
| **S10** | 0.164 | 0.516 | 0.534 | 0.411 | 0 |
| **S11** | 0.252 | 0 | 0.531 | 0.282 | 0.435 |
| **S12** | 0.156 | 0.268 | 0.169 | 0.210 | 0.271 |
| **S13** | 0.074 | 0.583 | 0.582 | 0.226 | 0.400 |
| **S14** | 0.567 | 0.686 | 0.685 | 0.538 | 0.521 |
| **S15** | 0.492 | 0.703 | 0.660 | 0.598 | 0.469 |
| **S16** | 0.254 | 0.474 | 0.610 | 0.022 | 0.230 |
| **S17** | 0.170 | 0.189 | 0.560 | 0.310 | 0.493 |
| **S18** | 0.289 | 0.484 | 0.246 | 0.287 | 0.431 |
| **S19** | 0.079 | 0.067 | 0 | 0.016 | 0.258 |
| **S20** | 0.361 | 0.711 | 0.819 | 0.318 | 0.528 |
| **S21** | 0.196 | 0.123 | 0.370 | 0.368 | 0.527 |
| **S22** | 0.412 | 0.358 | 0.192 | 0.147 | 0.300 |
| **S23** | 0.373 | 0.432 | 0.543 | 0.052 | 0.497 |
| **S24** | 0.319 | 0.147 | 0.431 | 0.379 | 0.546 |

P values < 0.001 for all categories comparing to 0 MCC.

Supplementary Table 2: Modeling performances (MCC values) of the double-layer multi-temporal resolution model across all subjects (n=24) and categories in the **Match Response** decoding case.

| **Subject ID** | **Animal** | **Building** | **Plant** | **Tool** | **Vehicle** |
| --- | --- | --- | --- | --- | --- |
| **S1** | 0.368 | 0.147 | 0.536 | 0.380 | 0.265 |
| **S2** | 0.300 | 0.393 | 0.552 | 0.509 | 0.490 |
| **S3** | 0.603 | 0.572 | 0.674 | 0.556 | 0.601 |
| **S4** | 0.231 | 0.367 | 0.390 | 0.154 | 0.179 |
| **S5** | 0.544 | 0.461 | 0.375 | 0.319 | 0.508 |
| **S6** | 0.437 | 0.465 | 0.590 | 0.629 | 0.367 |
| **S7** | 0.347 | 0.583 | 0.549 | 0.468 | 0.308 |
| **S8** | 0.160 | 0.490 | 0.491 | 0.506 | 0.258 |
| **S9** | 0.598 | 0.416 | 0.578 | 0.302 | 0.492 |
| **S10** | 0.050 | 0.578 | 0.551 | 0.518 | 0.015 |
| **S11** | 0.227 | 0 | 0.267 | 0.429 | 0.132 |
| **S12** | 0.241 | 0.299 | 0.413 | 0.249 | 0.332 |
| **S13** | 0.547 | 0.583 | 0.518 | 0.569 | 0.589 |
| **S14** | 0.485 | 0.687 | 0.727 | 0.544 | 0.694 |
| **S15** | 0.441 | 0.676 | 0.705 | 0.560 | 0.402 |
| **S16** | 0.611 | 0.170 | 0.236 | 0.392 | 0.347 |
| **S17** | 0.514 | 0.610 | 0.525 | 0.861 | 0.411 |
| **S18** | 0.152 | 0.314 | 0.406 | 0.192 | 0.431 |
| **S19** | 0.188 | 0.159 | 0.245 | 0.200 | 0.333 |
| **S20** | 0.610 | 0.610 | 0.749 | 0.700 | 0.743 |
| **S21** | 0.372 | 0.476 | 0.508 | 0.261 | 0.103 |
| **S22** | 0.184 | 0.252 | 0.394 | 0.082 | 0 |
| **S23** | 0.447 | 0.464 | 0.727 | 0.414 | 0.461 |
| **S24** | 0.412 | 0.461 | 0.322 | 0.414 | 0.215 |

P values < 0.001 for all categories comparing to 0 MCC.

Supplementary Table 3: Modeling performances (Informedness) of the double-layer multi-temporal resolution model across all subjects (n=24) and categories in the **Sample Response** decoding case.

| **Subject ID** | **Animal** | **Building** | **Plant** | **Tool** | **Vehicle** |
| --- | --- | --- | --- | --- | --- |
| **S1** | 0.180 | 0.045 | 0.414 | 0.217 | 0.312 |
| **S2** | 0.214 | 0.268 | 0.509 | 0.194 | 0.398 |
| **S3** | 0.262 | 0.588 | 0.585 | 0.247 | 0.383 |
| **S4** | 0.126 | 0.340 | 0.467 | 0.067 | 0.129 |
| **S5** | 0.131 | 0.187 | 0.353 | 0.635 | 0.538 |
| **S6** | 0.616 | 0.689 | 0.498 | 0.322 | 0.587 |
| **S7** | 0.255 | 0.341 | 0.323 | 0.189 | 0.576 |
| **S8** | 0.112 | 0.283 | 0.516 | 0.235 | 0.176 |
| **S9** | 0.314 | 0.450 | 0.285 | 0.297 | 0.066 |
| **S10** | 0.110 | 0.448 | 0.501 | 0.348 | 0 |
| **S11** | 0.200 | 0 | 0.498 | 0.207 | 0.380 |
| **S12** | 0.134 | 0.250 | 0.155 | 0.203 | 0.251 |
| **S13** | 0.051 | 0.450 | 0.473 | 0.165 | 0.289 |
| **S14** | 0.498 | 0.583 | 0.622 | 0.420 | 0.376 |
| **S15** | 0.399 | 0.611 | 0.556 | 0.524 | 0.417 |
| **S16** | 0.232 | 0.437 | 0.580 | 0.016 | 0.195 |
| **S17** | 0.142 | 0.150 | 0.464 | 0.219 | 0.368 |
| **S18** | 0.275 | 0.477 | 0.205 | 0.249 | 0.369 |
| **S19** | 0.055 | 0.033 | 0 | 0.013 | 0.173 |
| **S20** | 0.214 | 0.592 | 0.754 | 0.228 | 0.392 |
| **S21** | 0.187 | 0.108 | 0.349 | 0.368 | 0.478 |
| **S22** | 0.336 | 0.317 | 0.156 | 0.125 | 0.267 |
| **S23** | 0.320 | 0.408 | 0.473 | 0.051 | 0.443 |
| **S24** | 0.274 | 0.125 | 0.410 | 0.355 | 0.487 |

P values < 0.001 for all categories compared to 0 Informedness.

Supplementary Table 4: Modeling performances (markedness) of the double-layer multi-temporal resolution model across all subjects (n=24) and categories in the **Sample Response** decoding case.

| **Subject ID** | **Animal** | **Building** | **Plant** | **Tool** | **Vehicle** |
| --- | --- | --- | --- | --- | --- |
| **S1** | 0.156 | 0.058 | 0.407 | 0.337 | 0.458 |
| **S2** | 0.420 | 0.444 | 0.614 | 0.273 | 0.513 |
| **S3** | 0.496 | 0.681 | 0.726 | 0.370 | 0.648 |
| **S4** | 0.166 | 0.397 | 0.515 | 0.153 | 0.141 |
| **S5** | 0.385 | 0.291 | 0.545 | 0.692 | 0.814 |
| **S6** | 0.842 | 0.810 | 0.556 | 0.339 | 0.627 |
| **S7** | 0.569 | 0.643 | 0.549 | 0.398 | 0.804 |
| **S8** | 0.178 | 0.476 | 0.626 | 0.557 | 0.281 |
| **S9** | 0.567 | 0.597 | 0.302 | 0.396 | 0.071 |
| **S10** | 0.244 | 0.593 | 0.570 | 0.485 | 0 |
| **S11** | 0.317 | 0 | 0.566 | 0.383 | 0.498 |
| **S12** | 0.182 | 0.288 | 0.183 | 0.216 | 0.293 |
| **S13** | 0.107 | 0.756 | 0.715 | 0.308 | 0.554 |
| **S14** | 0.645 | 0.808 | 0.755 | 0.689 | 0.722 |
| **S15** | 0.607 | 0.809 | 0.782 | 0.684 | 0.528 |
| **S16** | 0.280 | 0.515 | 0.641 | 0.032 | 0.271 |
| **S17** | 0.203 | 0.239 | 0.676 | 0.438 | 0.661 |
| **S18** | 0.304 | 0.491 | 0.297 | 0.331 | 0.505 |
| **S19** | 0.113 | 0.138 | 0 | 0.021 | 0.386 |
| **S20** | 0.607 | 0.854 | 0.890 | 0.444 | 0.790 |
| **S21** | 0.206 | 0.138 | 0.393 | 0.368 | 0.580 |
| **S22** | 0.506 | 0.405 | 0.236 | 0.174 | 0.337 |
| **S23** | 0.434 | 0.456 | 0.624 | 0.051 | 0.558 |
| **S24** | 0.371 | 0.173 | 0.452 | 0.404 | 0.614 |

P values < 0.001 for all categories compared to 0 markedness.

Supplementary Table 5: Modeling performances (Informedness) of the double-layer multi-temporal resolution model across all subjects (n=24) and categories in the **Match Response** decoding case.

| **Subject ID** | **Animal** | **Building** | **Plant** | **Tool** | **Vehicle** |
| --- | --- | --- | --- | --- | --- |
| **S1** | 0.404 | 0.125 | 0.519 | 0.239 | 0.156 |
| **S2** | 0.247 | 0.342 | 0.522 | 0.461 | 0.431 |
| **S3** | 0.468 | 0.511 | 0.554 | 0.455 | 0.523 |
| **S4** | 0.211 | 0.350 | 0.383 | 0.116 | 0.160 |
| **S5** | 0.439 | 0.333 | 0.302 | 0.254 | 0.413 |
| **S6** | 0.374 | 0.424 | 0.554 | 0.570 | 0.296 |
| **S7** | 0.271 | 0.425 | 0.506 | 0.382 | 0.218 |
| **S8** | 0.105 | 0.417 | 0.446 | 0.383 | 0.193 |
| **S9** | 0.589 | 0.399 | 0.550 | 0.293 | 0.453 |
| **S10** | 0.030 | 0.539 | 0.510 | 0.439 | 0.010 |
| **S11** | 0.204 | 0 | 0.256 | 0.355 | 0.120 |
| **S12** | 0.197 | 0.283 | 0.410 | 0.194 | 0.283 |
| **S13** | 0.377 | 0.450 | 0.429 | 0.457 | 0.503 |
| **S14** | 0.360 | 0.558 | 0.623 | 0.449 | 0.618 |
| **S15** | 0.321 | 0.546 | 0.617 | 0.453 | 0.296 |
| **S16** | 0.530 | 0.170 | 0.198 | 0.399 | 0.326 |
| **S17** | 0.368 | 0.483 | 0.464 | 0.778 | 0.316 |
| **S18** | 0.117 | 0.290 | 0.371 | 0.189 | 0.369 |
| **S19** | 0.120 | 0.108 | 0.174 | 0.136 | 0.302 |
| **S20** | 0.484 | 0.508 | 0.632 | 0.538 | 0.635 |
| **S21** | 0.245 | 0.450 | 0.455 | 0.236 | 0.091 |
| **S22** | 0.150 | 0.142 | 0.375 | 0.071 | 0 |
| **S23** | 0.375 | 0.425 | 0.623 | 0.350 | 0.333 |
| **S24** | 0.336 | 0.383 | 0.296 | 0.350 | 0.187 |

P values < 0.001 for all categories compared to 0 Informedness.

Supplementary Table 6: Modeling performances (markedness) of the double-layer multi-temporal resolution model across all subjects (n=24) and categories in the **Match Response** decoding case.

| **Subject ID** | **Animal** | **Building** | **Plant** | **Tool** | **Vehicle** |
| --- | --- | --- | --- | --- | --- |
| **S1** | 0.335 | 0.174 | 0.553 | 0.605 | 0.449 |
| **S2** | 0.364 | 0.452 | 0.583 | 0.561 | 0.556 |
| **S3** | 0.777 | 0.640 | 0.818 | 0.680 | 0.690 |
| **S4** | 0.253 | 0.384 | 0.396 | 0.205 | 0.200 |
| **S5** | 0.674 | 0.638 | 0.467 | 0.399 | 0.625 |
| **S6** | 0.511 | 0.510 | 0.628 | 0.694 | 0.455 |
| **S7** | 0.444 | 0.801 | 0.597 | 0.573 | 0.435 |
| **S8** | 0.243 | 0.577 | 0.541 | 0.668 | 0.346 |
| **S9** | 0.607 | 0.433 | 0.607 | 0.311 | 0.534 |
| **S10** | 0.082 | 0.620 | 0.595 | 0.612 | 0.022 |
| **S11** | 0.252 | 0 | 0.278 | 0.519 | 0.144 |
| **S12** | 0.296 | 0.316 | 0.417 | 0.319 | 0.389 |
| **S13** | 0.794 | 0.756 | 0.625 | 0.708 | 0.690 |
| **S14** | 0.652 | 0.846 | 0.848 | 0.659 | 0.780 |
| **S15** | 0.606 | 0.837 | 0.806 | 0.692 | 0.545 |
| **S16** | 0.705 | 0.170 | 0.280 | 0.385 | 0.368 |
| **S17** | 0.717 | 0.770 | 0.594 | 0.953 | 0.533 |
| **S18** | 0.197 | 0.339 | 0.445 | 0.195 | 0.505 |
| **S19** | 0.294 | 0.234 | 0.344 | 0.295 | 0.366 |
| **S20** | 0.769 | 0.734 | 0.887 | 0.910 | 0.870 |
| **S21** | 0.564 | 0.502 | 0.567 | 0.289 | 0.115 |
| **S22** | 0.226 | 0.449 | 0.413 | 0.095 | 0 |
| **S23** | 0.534 | 0.506 | 0.848 | 0.490 | 0.638 |
| **S24** | 0.506 | 0.554 | 0.350 | 0.490 | 0.246 |

P values < 0.001 for all categories compared to 0 markedness.

Supplementary Table 7: Modeling performances (MCC values) of the double-layer multi-temporal resolution model across all subjects (n=24) and categories in the **Time-shifted** control case.

| **Subject ID** | **Animal** | **Building** | **Plant** | **Tool** | **Vehicle** |
| --- | --- | --- | --- | --- | --- |
| **S1** | 0 | 0 | 0 | 0 | 0 |
| **S2** | 0 | 0.086 | 0.272 | 0.155 | 0 |
| **S3** | 0 | 0.029 | 0 | 0 | 0 |
| **S4** | 0.028 | 0.073 | 0 | 0.027 | 0.002 |
| **S5** | 0.106 | 0.015 | 0 | 0.074 | 0 |
| **S6** | 0 | 0 | 0 | 0 | 0 |
| **S7** | 0 | 0.119 | 0 | 0 | 0.002 |
| **S8** | 0.245 | 0.115 | 0.027 | 0 | 0.261 |
| **S9** | 0 | 0 | 0 | 0 | 0 |
| **S10** | 0.183 | 0.006 | 0.024 | 0 | 0 |
| **S11** | 0 | 0 | 0 | 0.141 | 0 |
| **S12** | 0 | 0 | 0.105 | 0.135 | 0 |
| **S13** | 0.082 | 0 | 0.135 | 0 | 0.071 |
| **S14** | 0.035 | 0 | 0 | 0 | 0.039 |
| **S15** | 0 | 0 | 0 | 0 | 0.156 |
| **S16** | 0 | 0.144 | 0.017 | 0.068 | 0 |
| **S17** | 0.019 | 0.065 | 0 | 0 | 0 |
| **S18** | 0.174 | 0.077 | 0 | 0.058 | 0 |
| **S19** | 0.016 | 0.222 | 0 | 0 | 0 |
| **S20** | 0.263 | 0 | 0.102 | 0 | 0 |
| **S21** | 0.086 | 0 | 0 | 0 | 0 |
| **S22** | 0.181 | 0 | 0 | 0 | 0.086 |
| **S23** | 0.070 | 0.051 | 0 | 0.106 | 0.043 |
| **S24** | 0.013 | 0 | 0.080 | 0.170 | 0 |

P value = 0.0014, 0.0024, 0.0253, 0.0037, 0.0422 compared to 0 MCC for the categories of Animal, Building, Plant, Tool, and Vehicle, respectively.

**Supplementary Material 4: Surrogate Spike Trains Controls for Model Validation**

**Rationale**: To further validate the robustness of the multi-temporal-resolution decoding model and ensure that its performance is not driven by overfitting or spurious correlations, we conducted two additional surrogate-based control analyses. Unlike the Time-Shifted Control and the Label-Shuffled Control described in the main manuscript—which were used for both model training and testing—these new controls exclusively test the model’s ability to classify category labels using surrogate spike train inputs to assess the importance of precise spatiotemporal structure in classification.

**Data**: The same twenty-four subjects included in the main analysis. The decoding task involved the same five memory categories (Animal, Building, Plant, Tool, Vehicle) and two decoding conditions: Sample Response and Match Response. For each decoding condition, the corresponding trained multi-temporal-resolution model was used.

**Methods**:

**Control 1 – Circular Time Shift Per Neuron:** For each neuron at each trial, its spike train was circularly shifted by a random time offset unique to that neuron, with wrapping around the trial duration. This procedure preserves the spike count, inter spike interval distribution, and firing rate, but disrupts temporal coordination across neurons.

**Control 2 – Random Jitter of Spike Times:** For each neuron at each trial, each spike was jittered randomly at the range of -20 to +20 milliseconds. This procedure breaks the temporal code of each neuronal spiking activities to different extents while preserving the overall firing rate.

For all controls, the surrogate spike trains were generated 100 times. The trained multi-temporal-resolution models were then tested on these surrogate datasets, and the Matthews correlation coefficient (MCC) was calculated for each iteration.

*Statistical Comparison*: One-sample t-test was used to determine whether such MCCs across subjects (n = 24) for each category (c = 5) at each control case was significantly greater than zero (chance level). Additionally, Paired t-tests were also used to compare each control model with the multi-temporal-resolution model, using matched subject-category pairs.

**Results**: Performance decreased substantially when surrogate spike trains were used as inputs (Supplementary Tables 8–11), confirming the importance of intact spatiotemporal coding for decoding. In both controls, MCCs dropped to near-zero levels largely across subjects and categories, indicating the model could not decode category information without temporally coordinated neural activity. Paired comparisons revealed significantly lower performance than with the original spike data (p < 0.001 for all comparisons).

Supplementary Table 8: Mean decoding performance (MCC) of the trained double-layer multi-temporal resolution model across all subjects (n = 24) and categories in the *Sample Response* condition, using surrogate spike trains generated from **Control 1 – Circular Time Shift Per Neuron**. In Supplementary Tables 8-11, significance indicators *, **, and *** denote that the corresponding real-data MCC value exceeds the 90th, 95th, and 99th percentile of the 100 surrogate distributions, respectively.

| **Subject ID** | **Animal** | **Building** | **Plant** | **Tool** | **Vehicle** |
| --- | --- | --- | --- | --- | --- |
| **S1** | 0.085 * | 0.129 | 0.098 *** | 0 *** | 0.222 * |
| **S2** | -0.018 *** | 0.048 *** | 0.077 *** | -0.023 *** | 0.009 *** |
| **S3** | 0 *** | 0.056 *** | 0.090 *** | -0.026 *** | -0.035 *** |
| **S4** | 0.050 * | 0.106 *** | 0.045 *** | -0.050 *** | -0.010 *** |
| **S5** | 0.033 * | 0.140 * | 0.144 *** | -0.026 *** | 0.053 *** |
| **S6** | 0.092 *** | 0.055 *** | 0.087 *** | -0.044 *** | 0.037 *** |
| **S7** | -0.006 *** | 0.034 *** | 0.065 *** | 0.008 *** | 0.048 *** |
| **S8** | -0.019 ** | 0.064 *** | 0.062 *** | -0.016 *** | 0.027 ** |
| **S9** | 0.036 *** | 0.002 *** | 0.034 *** | 0.005 *** | 0.039 |
| **S10** | -0.014 ** | 0.072 *** | 0.049 *** | -0.055 *** | -0.049 |
| **S11** | 0.006 *** | -0.057 | 0.030 *** | -0.007 *** | 0.007 *** |
| **S12** | -0.011 ** | 0.022 *** | 0.038 ** | 0.057 *** | -0.038 *** |
| **S13** | -0.015 | 0.002 *** | 0.016 *** | 0.004 *** | 0.026 *** |
| **S14** | 0.044 *** | 0.023 *** | 0.063 *** | 0.027 *** | 0.034 *** |
| **S15** | -0.030 *** | 0.074 *** | 0.026 *** | 0.042 *** | 0.043 *** |
| **S16** | 0.034 * | 0.115 *** | 0.008 *** | -0.004 | 0.060 * |
| **S17** | 0 *** | 0.034 ** | 0.036 *** | 0.019 *** | 0.070 *** |
| **S18** | 0.041 *** | 0.012 *** | -0.055 *** | -0.037 *** | 0.115 *** |
| **S19** | -0.037 * | 0.004 | 0 | -0.053 | -0.015 *** |
| **S20** | -0.008 *** | 0.047 *** | 0.031 *** | 0.014 *** | 0.018 *** |
| **S21** | -0.019 *** | 0.058 | 0.074 *** | -0.006 *** | 0.024 *** |
| **S22** | 0.009 *** | 0.074 *** | 0.060 ** | -0.009 ** | -0.003 *** |
| **S23** | -0.016 *** | 0.008 *** | 0.035 *** | -0.052 * | 0.015 *** |
| **S24** | -0.003 *** | -0.003 *** | 0.089 *** | 0.059 *** | 0.026 *** |

Note that MCC value < 0.0001 was presented as 0 for simplicity.

P value = 0.18, <0.001, <0.001, 0.29, 0.013 compared to 0 MCC for the categories of Animal, Building, Plant, Tool, and Vehicle, respectively.

P values < 0.001 for all categories compared to MCC yield from the double-layer multi-temporal resolution model.

Supplementary Table 9: Mean decoding performance (MCC) of the trained double-layer multi-temporal resolution model across all subjects (n = 24) and categories in the *Sample Response* condition, using surrogate spike trains generated from **Control 2 – Random Jitter of Spike Times**.

| **Subject ID** | **Animal** | **Building** | **Plant** | **Tool** | **Vehicle** |
| --- | --- | --- | --- | --- | --- |
| **S1** | 0.021 *** | -0.084 *** | 0.012 *** | 0.005 *** | 0.034 *** |
| **S2** | 0 *** | 0.010 *** | 0 *** | 0.030 *** | 0 *** |
| **S3** | -0.033 *** | 0 *** | 0.024 *** | -0.049 *** | 0.002 *** |
| **S4** | 0.001 *** | 0 *** | 0 *** | -0.171 *** | -0.026 *** |
| **S5** | 0 *** | 0 *** | 0 *** | 0.012 *** | 0 *** |
| **S6** | 0 *** | 0.018 *** | 0 *** | 0.003 *** | 0 *** |
| **S7** | 0.005 *** | -0.007 *** | -0.007 *** | 0.049 *** | -0.027 *** |
| **S8** | -0.003 *** | 0 *** | 0 *** | -0.044 *** | 0.165 *** |
| **S9** | 0.042 *** | 0.049 *** | 0.218 *** | -0.096 *** | -0.143 *** |
| **S10** | -0.044 *** | 0.043 *** | 0.147 *** | -0.115 *** | -0.178 *** |
| **S11** | 0 *** | 0.097 | 0 *** | 0.007 *** | 0 *** |
| **S12** | 0.017 *** | 0.035 *** | -0.053 *** | 0.019 *** | 0.040 *** |
| **S13** | 0 *** | 0.025 *** | 0 *** | -0.023 *** | 0 *** |
| **S14** | 0 *** | 0.020 *** | 0 *** | -0.009 *** | 0 *** |
| **S15** | 0 *** | 0 *** | 0 *** | 0 *** | 0 *** |
| **S16** | 0.083 *** | 0.113 *** | 0.093 *** | -0.081 *** | 0.016 *** |
| **S17** | 0.005 *** | 0.076 *** | 0 *** | 0 *** | 0 *** |
| **S18** | 0.024 *** | -0.025 *** | -0.048 *** | 0.016 *** | 0 *** |
| **S19** | 0.076 | 0.022 *** | 0 | -0.111 *** | -0.051 *** |
| **S20** | 0 *** | 0 *** | 0 *** | 0 *** | 0 *** |
| **S21** | 0.010 *** | 0 *** | 0.002 *** | 0.003 *** | 0.148 *** |
| **S22** | 0.035 *** | -0.050 *** | 0.008 *** | 0.009 *** | 0.106 *** |
| **S23** | 0 *** | 0 *** | 0.100 *** | -0.056 *** | 0 *** |
| **S24** | -0.019 *** | 0.010 *** | -0.190 *** | 0.023 *** | 0.011 *** |

Note that MCC value < 0.0001 was presented as 0 for simplicity.

P value = 0.13, 0.10, 0.41, 0.04, 0.78 compared to 0 MCC for the categories of Animal, Building, Plant, Tool, and Vehicle, respectively.

P values < 0.001 for all categories compared to MCC yield from the double-layer multi-temporal resolution model.

Supplementary Table 10: Mean decoding performance (MCC) of the trained double-layer multi-temporal resolution model across all subjects (n = 24) and categories in the *Match Response* condition, using surrogate spike trains generated from **Control 1 – Circular Time Shift Per Neuron**.

| **Subject ID** | **Animal** | **Building** | **Plant** | **Tool** | **Vehicle** |
| --- | --- | --- | --- | --- | --- |
| **S1** | 0.106 *** | 0.072 | 0.014 *** | 0.018 *** | 0.112 |
| **S2** | -0.010 *** | 0.007 *** | 0.049 *** | 0.040 *** | -0.037 *** |
| **S3** | -0.002 *** | 0.008 *** | 0.070 *** | -0.018 *** | 0.012 *** |
| **S4** | 0.024 *** | 0.054 *** | 0.088 *** | -0.008 ** | 0.007 *** |
| **S5** | 0.009 *** | 0.054 *** | 0.029 *** | -0.016 *** | -0.002 *** |
| **S6** | 0.007 *** | 0.107 *** | 0.016 *** | -0.007 *** | 0.050 *** |
| **S7** | -0.022 *** | -0.002 *** | 0.035 *** | -0.012 *** | 0.032 *** |
| **S8** | -0.028 *** | 0.031 *** | 0.046 *** | -0.030 *** | 0.016 *** |
| **S9** | -0.060 *** | 0.009 *** | -0.002 *** | 0.040 *** | -0.029 *** |
| **S10** | -0.032 * | 0.095 *** | -0.011 *** | 0.018 *** | -0.068 * |
| **S11** | -0.036 *** | -0.010 | 0.002 *** | 0.102 *** | -0.012 ** |
| **S12** | 0.027 *** | 0.068 *** | 0.013 *** | -0.033 *** | -0.028 *** |
| **S13** | -0.017 *** | 0.053 *** | 0.059 *** | 0.037 *** | 0.003 *** |
| **S14** | 0.007 *** | 0.062 *** | 0.080 *** | -0.019 *** | 0.052 *** |
| **S15** | -0.024 *** | -0.024 *** | 0.020 *** | 0.016 *** | 0.030 *** |
| **S16** | 0.045 *** | 0.059 | 0.039 *** | -0.022 *** | 0.005 *** |
| **S17** | 0 *** | -0.023 *** | 0.059 *** | -0.013 *** | -0.019 *** |
| **S18** | -0.060 *** | -0.031 *** | 0.014 *** | 0.006 *** | -0.016 *** |
| **S19** | -0.015 *** | -0.050 *** | 0 *** | -0.044 *** | 0.018 *** |
| **S20** | 0.012 *** | 0.048 *** | 0.063 *** | -0.001 *** | 0.025 *** |
| **S21** | 0.037 *** | 0.026 *** | 0.039 *** | 0.036 *** | -0.013 * |
| **S22** | 0.024 *** | 0.007 *** | 0.056 *** | -0.037 * | 0.005 |
| **S23** | -0.014 *** | 0.053 *** | 0.056 *** | 0.054 *** | -0.035 *** |
| **S24** | -0.011 *** | 0.242 ** | 0.113 *** | -0.058 *** | -0.019 *** |

Note that MCC value < 0.0001 was presented as 0 for simplicity.

P value = 0.85, 0.005, <0.001, 0.80, 0.62 compared to 0 MCC for the categories of Animal, Building, Plant, Tool, and Vehicle, respectively.

P values < 0.001 for all categories compared to MCC yield from the double-layer multi-temporal resolution model.

Supplementary Table 11: Mean decoding performance (MCC) of the trained double-layer multi-temporal resolution model across all subjects (n = 24) and categories in the *Match Response* condition, using surrogate spike trains generated from **Control 2 – Random Jitter of Spike Times**.

| **Subject ID** | **Animal** | **Building** | **Plant** | **Tool** | **Vehicle** |
| --- | --- | --- | --- | --- | --- |
| **S1** | 0.070 *** | 0.028 *** | 0 *** | 0.070 *** | -0.126 *** |
| **S2** | 0.002 *** | 0 *** | 0 *** | 0.004 *** | 0 *** |
| **S3** | 0.013 *** | 0.021 *** | 0.001 *** | -0.008 *** | 0.019 *** |
| **S4** | 0 *** | 0 *** | 0 *** | 0.178 | 0.072 *** |
| **S5** | 0 *** | 0 *** | 0 *** | 0 *** | 0 *** |
| **S6** | -0.086 *** | 0.021 *** | 0.018 *** | 0 *** | 0 *** |
| **S7** | 0.012 *** | -0.117 *** | -0.069 *** | -0.012 *** | -0.011 *** |
| **S8** | 0 *** | 0 *** | 0 *** | -0.030 *** | 0 *** |
| **S9** | 0.048 *** | 0.049 *** | 0.108 *** | -0.099 *** | -0.143 *** |
| **S10** | 0.016 | 0 *** | 0.045 *** | 0.060 *** | 0.161 |
| **S11** | 0.185 *** | -0.078 *** | 0 *** | 0 *** | 0.068 *** |
| **S12** | -0.002 *** | 0.030 *** | 0.050 *** | -0.018 *** | 0.001 *** |
| **S13** | 0.018 *** | 0 *** | 0 *** | -0.026 *** | 0 *** |
| **S14** | 0 *** | -0.026 *** | 0.100 *** | -0.068 *** | -0.047 *** |
| **S15** | -0.020 *** | 0.006 *** | 0 *** | 0 *** | 0 *** |
| **S16** | 0 *** | 0.063 *** | 0.009 *** | 0.099 *** | -0.009 *** |
| **S17** | 0 *** | 0 *** | 0 *** | 0.052 *** | 0 *** |
| **S18** | -0.125 *** | -0.030 *** | 0.056 *** | 0.061 *** | 0.054 *** |
| **S19** | -0.083 *** | 0.003 *** | 0 *** | -0.058 *** | -0.141 *** |
| **S20** | 0.005 *** | -0.065 *** | 0 *** | -0.052 *** | 0.021 *** |
| **S21** | 0 *** | 0 *** | -0.007 *** | 0.036 *** | -0.017 *** |
| **S22** | -0.016 *** | 0.033 *** | 0 *** | 0.048 *** | 0.002 |
| **S23** | 0.050 *** | 0 *** | 0 *** | 0.096 *** | -0.120 *** |
| **S24** | 0.030 *** | -0.104 *** | 0.048 *** | 0.072 *** | 0.031 *** |

Note that MCC value < 0.0001 was presented as 0 for simplicity.

P value = 0.68, 0.46, 0.06, 0.2, 0.59 compared to 0 MCC for the categories of Animal, Building, Plant, Tool, and Vehicle, respectively.

P values < 0.001 for all categories compared to MCC yield from the double-layer multi-temporal resolution model.

**Supplementary Material 5: Comparison to Rate-coding Based Models**

**Rationale**: Rate-coding models typically decode memory representations by computing the average spike firing rate within fixed-duration time bins. These averaged rates are then used as input features for classification. To better evaluate the advantage of incorporating multi-scale temporal information, we compared the performance of our multi-temporal-resolution model with several rate-coding models using fixed bin sizes.

**Data**: The same twenty-four subjects included in the main analysis. The decoding task involved the same five memory categories (Animal, Building, Plant, Tool, Vehicle) and two decoding conditions: Sample Response and Match Response.

**Methods**: We implemented four rate-coding models, each using a different fixed bin size: 20 ms, 50 ms, 100 ms, and 2000 ms (the full decoding window). For each neuron, spike counts were computed within each bin, resulting in input vectors of the following lengths:

- 20 ms bins: 100 features per neuron
- 50 ms bins: 40 features per neuron
- 100 ms bins: 20 features per neuron
- 2000 ms bin: 1 feature per neuron (i.e., the firing rate over the full window)

The input vectors were concatenated across neurons to form the full feature set for decoding. The same modeling algorithm, cross-validation settings, and evaluation methods were used as the same as described in the main analysis.

It is worth noting that these rate-coding models are essentially “single-resolution” models as we described in the manuscript and compared in our previous study (She et al., 2022 *Neural Computation*). Each bin size corresponds to a specific temporal resolution, while the binning process also essentially corresponds to a zero^th^ B-spline, which is slightly different to the described single-resolution model, which used a third-order B-spline.

*Statistical Comparison*: One-sample t-test was used to determine whether MCCs across subjects (n = 24) for each category (c = 5) and for each bin size was significantly greater than zero (chance level). Paired t-tests were conducted to compare decoding performance between different bin sizes (e.g., MCC using 20 ms bins vs. 50 ms bins). Additionally, Paired t-tests were also used to compare each fixed-bin model with the multi-temporal-resolution model, using matched subject-category pairs.

**Results (Supplementary Figure 3)**:

First and foremost, multi-temporal-resolution model significantly outperformed all rate-coding based models across all bin sizes (p<0.001 in all paired comparisons).

Among the rate-coding models, those using bin sizes of 20 ms, 50 ms, and 100 ms produced decoding performances that were significantly above 0 MCC (chance level). Additionally, a general trend was observed in which smaller bin sizes yielded better performance, suggesting that finer temporal granularity improves decoding accuracy. Finally, the model using a single 2000 ms bin (i.e., the average firing rate over the entire decoding window) start yielding non-significantly different performance from chance.

Detailed MCC values and p-values from statistical comparisons are provided in the corresponding Supplementary Tables 12-19.


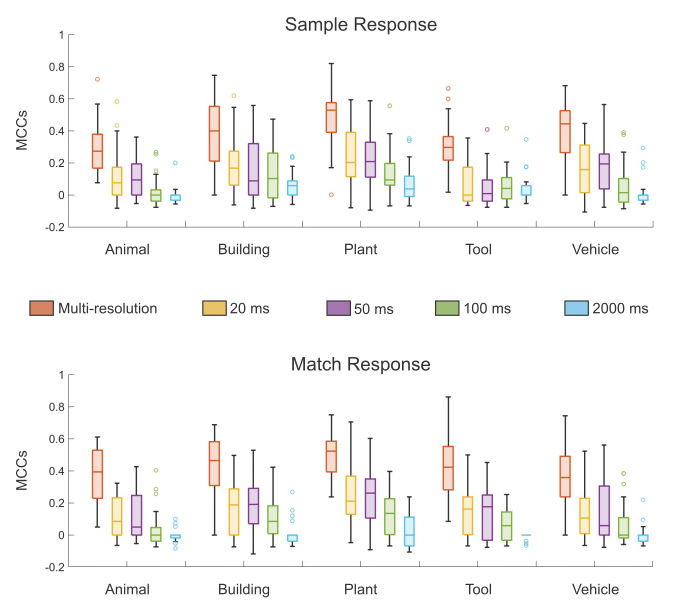


Supplementary Figure 3: Comparison of model performance in both decoding cases of Sample Response (top) and Match Response (bottom) across all subjects (n=24) and categories. The color of bars indicates the used bin size (temporal resolution) for generating the input features. Red: multi-temporal resolution in main analysis; yellow: 20 milliseconds; purple: 50 milliseconds; green: 100 milliseconds; blue: 2000 milliseconds (the entire decoding window).

Supplementary Table 12: Modeling performances (MCC values) of the **rate code-based model (bin size=20 ms)** across all subjects (n=24) and categories in the **Sample Response** decoding case.

| **Subject ID** | **Animal** | **Building** | **Plant** | **Tool** | **Vehicle** |
| --- | --- | --- | --- | --- | --- |
| **S1** | -0.074 | 0.215 | 0.048 | -0.042 | -0.025 |
| **S2** | 0.086 | -0.006 | 0.543 | 0 | 0.024 |
| **S3** | 0.065 | 0.342 | 0.444 | 0.059 | 0.376 |
| **S4** | 0 | 0.159 | 0.189 | -0.055 | 0.149 |
| **S5** | 0.157 | 0.113 | 0.074 | 0.214 | 0.153 |
| **S6** | 0.582 | 0.544 | 0.398 | 0.098 | 0.280 |
| **S7** | 0.103 | -0.042 | 0.254 | 0.059 | 0.445 |
| **S8** | 0.060 | 0.164 | 0.372 | 0.170 | 0.054 |
| **S9** | 0.356 | 0.252 | 0.175 | -0.039 | 0 |
| **S10** | -0.066 | -0.060 | 0.096 | -0.040 | -0.097 |
| **S11** | 0 | -0.062 | 0.383 | -0.058 | -0.039 |
| **S12** | 0 | 0.186 | 0.152 | 0.170 | 0.339 |
| **S13** | 0.100 | 0.238 | 0.307 | -0.038 | 0.102 |
| **S14** | 0.400 | 0.295 | 0.480 | 0.353 | 0.421 |
| **S15** | 0.433 | 0.619 | 0.541 | 0.175 | 0.345 |
| **S16** | 0 | 0 | -0.082 | 0 | -0.107 |
| **S17** | -0.053 | 0.200 | 0.147 | 0.246 | -0.068 |
| **S18** | 0.058 | 0.052 | 0.201 | 0 | 0.195 |
| **S19** | -0.037 | 0.087 | 0.202 | -0.055 | 0.114 |
| **S20** | 0.142 | 0.400 | 0.592 | -0.032 | 0.163 |
| **S21** | 0.254 | 0.167 | 0.016 | 0.174 | 0.400 |
| **S22** | -0.085 | 0.435 | 0.130 | 0 | 0.239 |
| **S23** | 0.086 | 0.098 | 0.287 | -0.067 | 0.218 |
| **S24** | 0.186 | 0.068 | 0.026 | 0.246 | 0.193 |

P value = 0.004, <0.001, <0.001, 0.018, <0.001 compared to 0 MCC for the categories of Animal, Building, Plant, Tool, and Vehicle, respectively.

P values < 0.001 for all categories compared to MCC yield from the double-layer multi-temporal resolution model.

When comparing the case of 20 ms vs. 50 ms bin size: P = 0.76, 0.22, 0.48, 0.42, 0.68 for the categories of Animal, Building, Plant, Tool, and Vehicle, respectively.

When comparing the case of 20 ms vs. 100 ms bin size: P = 0.003, 0.16, 0.002, 0.85, 0.02 for the categories of Animal, Building, Plant, Tool, and Vehicle, respectively.

When comparing the case of 20 ms vs. 2000 ms bin size: P = 0.002, 0.002, <0.001, 0.42, <0.001 for the categories of Animal, Building, Plant, Tool, and Vehicle, respectively.

Supplementary Table 13: Modeling performances (MCC values) of the **rate code-based model (bin size=50 ms)** across all subjects (n=24) and categories in the **Sample Response** decoding case.

| **Subject ID** | **Animal** | **Building** | **Plant** | **Tool** | **Vehicle** |
| --- | --- | --- | --- | --- | --- |
| **S1** | 0 | -0.082 | 0.145 | 0 | 0 |
| **S2** | 0.028 | 0 | 0.586 | 0.091 | 0.054 |
| **S3** | 0.116 | 0.397 | 0.586 | -0.060 | 0.228 |
| **S4** | 0 | 0 | 0.204 | 0.056 | 0.149 |
| **S5** | 0.157 | 0.142 | 0.234 | 0.161 | 0.281 |
| **S6** | 0.305 | 0.473 | 0.448 | 0.015 | 0.292 |
| **S7** | 0.183 | 0.086 | -0.036 | 0.059 | 0.564 |
| **S8** | -0.053 | 0.048 | 0.209 | -0.078 | 0.197 |
| **S9** | 0.252 | 0.364 | 0.046 | -0.067 | -0.039 |
| **S10** | 0 | -0.043 | 0.196 | -0.057 | -0.079 |
| **S11** | 0.008 | -0.005 | 0.289 | -0.041 | 0.213 |
| **S12** | 0.100 | 0.087 | -0.096 | 0.057 | 0.102 |
| **S13** | 0 | 0.320 | 0.152 | 0 | 0 |
| **S14** | 0.360 | 0.557 | 0.439 | 0.138 | 0.218 |
| **S15** | 0.199 | 0.458 | 0.457 | 0.408 | 0.022 |
| **S16** | 0.270 | -0.052 | 0.128 | 0 | 0 |
| **S17** | 0 | -0.041 | 0.365 | 0.259 | 0.292 |
| **S18** | 0.083 | 0.092 | 0.066 | -0.040 | 0.487 |
| **S19** | -0.037 | 0 | -0.048 | -0.067 | 0.164 |
| **S20** | 0.142 | 0.319 | 0.255 | -0.038 | 0.251 |
| **S21** | 0 | 0.048 | 0.271 | 0.097 | 0.191 |
| **S22** | 0.266 | 0.179 | 0.257 | 0.081 | 0.171 |
| **S23** | -0.030 | 0.225 | 0.135 | 0 | 0.197 |
| **S24** | 0.186 | 0.048 | 0.093 | 0.153 | 0.258 |

P value = <0.001, <0.001, <0.001, 0.06, <0.001 compared to 0 MCC for the categories of Animal, Building, Plant, Tool, and Vehicle, respectively.

P values < 0.001 for all categories compared to MCC yield from the double-layer multi-temporal resolution model.

When comparing the case of 50 ms vs. 100 ms bin size: P = 0.002, 0.50, 0.007, 0.62, <0.001 for the categories of Animal, Building, Plant, Tool, and Vehicle, respectively.

When comparing the case of 50 ms vs. 2000 ms bin size: P = <0.001, 0.02, <0.001, 0.74, <0.001 for the category of Animal, Building, Plant, Tool, and Vehicle, respectively.

Supplementary Table 14: Modeling performances (MCC values) of the **rate code-based model (bin size=100 ms)** across all subjects (n=24) and categories in the **Sample Response** decoding case.

| **Subject ID** | **Animal** | **Building** | **Plant** | **Tool** | **Vehicle** |
| --- | --- | --- | --- | --- | --- |
| **S1** | -0.074 | -0.067 | 0.239 | -0.030 | -0.044 |
| **S2** | 0.010 | 0.135 | 0.555 | 0.129 | 0.047 |
| **S3** | -0.047 | 0.352 | 0.344 | 0.034 | -0.054 |
| **S4** | 0.051 | 0.192 | 0.097 | 0 | 0 |
| **S5** | -0.039 | -0.063 | 0.131 | 0.414 | 0.107 |
| **S6** | 0.127 | 0.319 | 0.296 | 0.046 | 0.024 |
| **S7** | 0 | 0.364 | 0.087 | 0.099 | 0.389 |
| **S8** | -0.038 | 0.178 | 0.071 | 0.175 | 0.093 |
| **S9** | 0.254 | 0.067 | 0.115 | -0.039 | 0 |
| **S10** | -0.038 | -0.043 | 0.155 | -0.040 | 0.178 |
| **S11** | 0.029 | 0 | 0.114 | 0.174 | 0.102 |
| **S12** | -0.076 | -0.041 | -0.002 | 0.097 | -0.068 |
| **S13** | 0 | 0.021 | -0.068 | 0.057 | 0.027 |
| **S14** | 0.266 | 0.418 | 0.368 | 0.106 | 0.102 |
| **S15** | 0 | 0.471 | 0.100 | 0.108 | -0.087 |
| **S16** | 0 | 0.042 | 0.069 | 0 | -0.047 |
| **S17** | 0 | 0 | 0.046 | 0.030 | -0.079 |
| **S18** | -0.039 | 0.236 | 0.052 | 0.007 | 0.074 |
| **S19** | 0 | -0.072 | 0.066 | -0.078 | 0 |
| **S20** | -0.077 | 0.177 | 0.382 | -0.054 | -0.040 |
| **S21** | 0.034 | 0.286 | 0.066 | 0.203 | -0.056 |
| **S22** | 0 | -0.058 | -0.001 | -0.021 | 0 |
| **S23** | 0.149 | 0.164 | 0.089 | -0.038 | 0.267 |
| **S24** | -0.038 | 0 | -0.046 | 0.057 | 0.376 |

P value = 0.32, 0.001, <0.001, 0.01, 0.06 compared to 0 MCC for the categories of Animal, Building, Plant, Tool, and Vehicle, respectively.

P values < 0.001 for all categories compared to MCC yield from the double-layer multi-temporal resolution model.

When comparing the case of 100 ms vs. 2000 ms bin size: P = 0.29, 0.05, 0.06, 0.45, 0.11 for the categories of Animal, Building, Plant, Tool, and Vehicle, respectively.

Supplementary Table 15: Modeling performances (MCC values) of the **rate code-based model (bin size=2000 ms)** across all subjects (n=24) and categories in the **Sample Response** decoding case.

| **Subject ID** | **Animal** | **Building** | **Plant** | **Tool** | **Vehicle** |
| --- | --- | --- | --- | --- | --- |
| **S1** | 0 | 0.054 | -0.022 | 0 | -0.036 |
| **S2** | 0 | 0.079 | 0.083 | 0 | -0.048 |
| **S3** | -0.033 | -0.041 | 0.337 | 0 | 0 |
| **S4** | -0.057 | 0.063 | 0.120 | 0 | 0 |
| **S5** | 0 | 0.085 | 0.036 | 0.067 | 0 |
| **S6** | -0.057 | 0.239 | 0.076 | 0 | -0.034 |
| **S7** | 0 | 0.086 | 0.109 | 0 | 0.202 |
| **S8** | 0 | 0.178 | -0.071 | 0 | 0 |
| **S9** | 0 | 0 | -0.048 | -0.039 | 0 |
| **S10** | 0 | 0.086 | 0 | 0.174 | -0.039 |
| **S11** | 0 | 0 | 0.194 | 0 | 0 |
| **S12** | 0 | 0 | -0.068 | 0.175 | 0 |
| **S13** | 0 | 0 | 0 | 0.175 | 0 |
| **S14** | 0 | 0.093 | 0.090 | 0 | 0 |
| **S15** | 0.200 | -0.061 | 0.238 | 0.346 | 0 |
| **S16** | -0.050 | 0.088 | 0 | 0 | -0.047 |
| **S17** | -0.053 | 0.087 | 0.038 | -0.055 | -0.039 |
| **S18** | 0 | -0.041 | 0 | 0 | 0.035 |
| **S19** | 0 | -0.059 | -0.048 | -0.039 | 0 |
| **S20** | -0.054 | 0.232 | 0.212 | 0.047 | -0.057 |
| **S21** | -0.038 | 0 | 0.026 | 0 | 0 |
| **S22** | 0 | 0 | 0.350 | 0 | 0.171 |
| **S23** | 0 | 0.084 | -0.068 | 0.082 | 0.292 |
| **S24** | 0.034 | -0.041 | 0.111 | 0 | 0.027 |

P value = 0.660, 0.007, 0.009, 0.050, 0.311 compared to 0 MCC for the categories of Animal, Building, Plant, Tool, and Vehicle, respectively.

P values < 0.001 for all categories compared to MCC yield from the double-layer multi-temporal resolution model.

Supplementary Table 16: Modeling performances (MCC values) of the **rate code-based model (bin size=20 ms)** across all subjects (n=24) and categories in the **Match Response** decoding case.

| **Subject ID** | **Animal** | **Building** | **Plant** | **Tool** | **Vehicle** |
| --- | --- | --- | --- | --- | --- |
| **S1** | 0 | -0.047 | 0.145 | -0.030 | -0.036 |
| **S2** | 0.040 | 0.238 | 0.372 | 0.184 | 0.329 |
| **S3** | 0.065 | 0.209 | 0.211 | 0.338 | 0.144 |
| **S4** | 0 | 0.194 | 0.305 | 0.080 | 0.089 |
| **S5** | 0.321 | 0 | 0.153 | 0.031 | 0.189 |
| **S6** | -0.046 | 0.362 | 0.416 | 0.228 | 0.082 |
| **S7** | 0.259 | 0.251 | 0.285 | 0.208 | 0.008 |
| **S8** | 0.254 | 0.186 | 0.295 | -0.038 | 0 |
| **S9** | 0.204 | 0.200 | 0.369 | 0.499 | 0.009 |
| **S10** | -0.066 | 0.183 | -0.048 | 0.055 | 0.031 |
| **S11** | 0.310 | -0.076 | 0.166 | 0.037 | -0.039 |
| **S12** | 0.060 | 0.093 | 0.111 | 0.353 | 0.171 |
| **S13** | 0.186 | 0.030 | 0.324 | 0.347 | 0.381 |
| **S14** | 0.176 | 0.405 | 0.704 | 0.225 | 0.421 |
| **S15** | -0.039 | 0.495 | 0.366 | 0.178 | 0.283 |
| **S16** | 0.258 | -0.036 | 0.163 | -0.047 | -0.067 |
| **S17** | 0.034 | 0.048 | 0.066 | 0.201 | 0.137 |
| **S18** | 0 | 0.337 | 0.071 | -0.070 | 0.092 |
| **S19** | 0 | -0.072 | 0.026 | -0.055 | 0.114 |
| **S20** | 0.275 | 0.322 | 0.212 | 0.142 | 0.521 |
| **S21** | 0.066 | -0.058 | 0.368 | 0.466 | 0 |
| **S22** | 0.100 | 0 | 0.164 | 0.057 | 0.054 |
| **S23** | 0.126 | 0.186 | 0.493 | -0.038 | 0.267 |
| **S24** | 0.209 | 0.326 | 0.029 | 0.248 | 0.115 |

P value <0.001 for all categories when compared to 0 MCC.

P values < 0.001 for all categories compared to MCC yield from the double-layer multi-temporal resolution model.

When comparing the case of 20 ms vs. 50 ms bin size: P = 0.91, 0.42, 0.99, 0.72, 0.64 for the categories of Animal, Building, Plant, Tool, and Vehicle, respectively.

When comparing the case of 20 ms vs. 100 ms bin size: P = 0.02, 0.16, 0.008, 0.05, 0.01 for the categories of Animal, Building, Plant, Tool, and Vehicle, respectively.

When comparing the case of 20 ms vs. 2000 ms bin size: P < 0.001 for all categories.

Supplementary Table 17: Modeling performances (MCC values) of the **rate code-based model (bin size=50 ms)** across all subjects (n=24) and categories in the **Match Response** decoding case.

| **Subject ID** | **Animal** | **Building** | **Plant** | **Tool** | **Vehicle** |
| --- | --- | --- | --- | --- | --- |
| **S1** | -0.042 | -0.118 | -0.094 | -0.052 | 0 |
| **S2** | 0.086 | 0.411 | 0.323 | 0.293 | 0.259 |
| **S3** | 0.145 | 0.199 | 0.421 | 0.269 | 0.408 |
| **S4** | 0.027 | -0.037 | 0.349 | -0.032 | -0.059 |
| **S5** | -0.039 | 0.174 | -0.009 | 0.214 | 0.059 |
| **S6** | 0.382 | 0.179 | 0.350 | 0.187 | 0.224 |
| **S7** | 0.036 | 0.125 | 0.009 | 0.179 | 0.056 |
| **S8** | 0.034 | 0.238 | 0.372 | 0.106 | 0.388 |
| **S9** | 0 | 0.294 | 0.350 | 0.245 | -0.078 |
| **S10** | 0 | 0.237 | -0.084 | -0.040 | -0.039 |
| **S11** | 0.098 | -0.088 | 0.194 | 0.304 | 0 |
| **S12** | 0 | -0.032 | -0.023 | -0.054 | 0.054 |
| **S13** | 0.179 | 0.228 | 0.516 | 0.097 | 0.311 |
| **S14** | 0.316 | 0.401 | 0.469 | -0.078 | 0.560 |
| **S15** | 0.093 | 0.412 | 0.246 | 0.452 | 0.498 |
| **S16** | 0.424 | 0.126 | 0.072 | 0.226 | 0.202 |
| **S17** | 0.312 | 0.444 | 0.135 | 0.275 | 0.054 |
| **S18** | -0.039 | 0.052 | 0.249 | 0.136 | 0.024 |
| **S19** | 0 | 0.087 | 0.272 | 0 | 0.311 |
| **S20** | 0.361 | 0.528 | 0.602 | 0.252 | 0.299 |
| **S21** | 0.060 | 0.124 | 0.304 | 0.181 | 0 |
| **S22** | -0.053 | -0.041 | 0.251 | -0.038 | 0 |
| **S23** | 0.316 | 0.201 | 0.322 | 0.170 | 0.174 |
| **S24** | 0 | 0.286 | 0.162 | -0.054 | -0.039 |

P value = 0.002, <0.001, <0.001, <0.001, <0.001 compared to 0 MCC for the categories of Animal, Building, Plant, Tool, and Vehicle, respectively.

P values < 0.001 for all categories compared to MCC yield from the double-layer multi-temporal resolution model.

When comparing the case of 50 ms vs. 100 ms bin size: P = 0.02, 0.03, 0.01, 0.02, 0.008 for the categories of Animal, Building, Plant, Tool, and Vehicle, respectively.

When comparing the case of 50 ms vs. 2000 ms bin size: P = 0.002, <0.001, <0.001, <0.001, <0.001 for the categories of Animal, Building, Plant, Tool, and Vehicle, respectively.

Supplementary Table 18: Modeling performances (MCC values) of the **rate code-based model (bin size=100 ms)** across all subjects (n=24) and categories in the **Match Response** decoding case.

| **Subject ID** | **Animal** | **Building** | **Plant** | **Tool** | **Vehicle** |
| --- | --- | --- | --- | --- | --- |
| **S1** | 0.057 | 0 | 0 | -0.030 | -0.044 |
| **S2** | 0.256 | 0.141 | 0.277 | 0.187 | -0.059 |
| **S3** | 0.145 | 0.423 | 0.228 | 0.096 | 0.014 |
| **S4** | 0 | 0.036 | 0.048 | 0.091 | -0.059 |
| **S5** | -0.039 | 0.082 | 0.288 | 0.113 | 0 |
| **S6** | -0.074 | 0.225 | 0.097 | -0.045 | 0.088 |
| **S7** | 0 | 0.102 | 0.221 | -0.038 | 0.056 |
| **S8** | -0.053 | 0.014 | 0.129 | -0.038 | 0.006 |
| **S9** | -0.054 | 0.047 | 0.129 | 0.057 | 0.097 |
| **S10** | 0 | 0.018 | 0.001 | -0.070 | -0.039 |
| **S11** | -0.040 | 0 | 0.138 | 0.193 | 0 |
| **S12** | -0.038 | -0.041 | 0.038 | -0.054 | -0.056 |
| **S13** | 0 | 0.186 | 0.167 | 0.057 | 0.383 |
| **S14** | 0.034 | 0.225 | -0.041 | 0.175 | 0.317 |
| **S15** | 0 | 0.267 | 0.168 | 0.029 | 0.238 |
| **S16** | 0.286 | -0.075 | 0 | 0.197 | 0.115 |
| **S17** | -0.053 | 0.413 | -0.002 | 0.246 | 0 |
| **S18** | 0 | 0.174 | -0.039 | 0.095 | 0 |
| **S19** | 0 | 0 | -0.068 | 0 | 0.164 |
| **S20** | 0.403 | 0.046 | 0.264 | 0.252 | 0.382 |
| **S21** | -0.038 | 0.087 | 0.147 | -0.007 | 0 |
| **S22** | 0.013 | 0 | 0.224 | -0.038 | 0 |
| **S23** | 0.066 | 0.134 | 0.395 | 0.057 | -0.039 |
| **S24** | -0.053 | 0.124 | 0.274 | 0 | 0 |

P value = 0.18, <0.001, <0.001, 0.005, 0.03 compared to 0 MCC for the categories of Animal, Building, Plant, Tool, and Vehicle, respectively.

P values < 0.001 for all categories compared to MCC yield from the double-layer multi-temporal resolution model.

When comparing the case of 100 ms vs. 2000 ms bin size: P = 0.13, 0.004, 0.002, 0.002, 0.04 for the categories of Animal, Building, Plant, Tool, and Vehicle, respectively.

Supplementary Table 19: Modeling performances (MCC values) of the **rate code-based model (bin size=2000 ms)** across all subjects (n=24) and categories in the **Match Response** decoding case.

| **Subject ID** | **Animal** | **Building** | **Plant** | **Tool** | **Vehicle** |
| --- | --- | --- | --- | --- | --- |
| **S1** | -0.042 | 0.119 | 0 | 0 | -0.025 |
| **S2** | 0 | 0 | 0.148 | 0 | -0.034 |
| **S3** | 0 | -0.011 | 0.186 | 0 | 0.219 |
| **S4** | 0 | -0.065 | 0.238 | 0 | 0 |
| **S5** | 0 | 0.118 | -0.107 | 0 | 0 |
| **S6** | 0.051 | 0.153 | -0.078 | 0 | -0.068 |
| **S7** | 0 | 0 | -0.069 | -0.054 | 0 |
| **S8** | 0 | -0.041 | 0.038 | 0 | 0.094 |
| **S9** | 0 | 0 | -0.097 | -0.039 | 0 |
| **S10** | -0.054 | 0 | 0 | 0 | -0.039 |
| **S11** | 0 | -0.062 | 0 | 0 | 0 |
| **S12** | 0 | -0.071 | 0 | 0 | 0 |
| **S13** | 0 | -0.058 | 0.130 | -0.038 | 0 |
| **S14** | -0.085 | -0.058 | 0.130 | 0 | -0.056 |
| **S15** | 0 | 0 | 0 | 0 | 0.050 |
| **S16** | 0.072 | 0 | 0 | -0.067 | 0 |
| **S17** | -0.053 | 0 | -0.083 | 0 | -0.068 |
| **S18** | 0.099 | 0 | -0.068 | 0 | 0 |
| **S19** | 0 | 0 | 0 | -0.055 | -0.040 |
| **S20** | 0 | 0 | 0.133 | 0 | 0 |
| **S21** | -0.053 | -0.041 | -0.001 | 0 | 0 |
| **S22** | 0 | 0 | -0.068 | 0 | 0 |
| **S23** | -0.038 | 0.087 | 0.090 | 0 | -0.056 |
| **S24** | 0 | 0.267 | -0.070 | 0 | -0.039 |

P value = 0.602, 0.401, 0.355, 0.025, 0.830 compared to 0 MCC for the categories of Animal, Building, Plant, Tool, and Vehicle, respectively.

P values < 0.001 for all categories compared to MCC yield from the double-layer multi-temporal resolution model.
